# Quantifying Landscape‐Flux via Single‐Cell Transcriptomics Uncovers the Underlying Mechanism of Cell Cycle

**DOI:** 10.1002/advs.202308879

**Published:** 2024-02-14

**Authors:** Ligang Zhu, Jin Wang

**Affiliations:** ^1^ College of Physics Jilin University Changchun 130021 P. R. China; ^2^ State Key Laboratory of Electroanalytical Chemistry Changchun Institute of Applied Chemistry Chinese Academy of Sciences Changchun 130022 P. R. China; ^3^ Center for Theoretical Interdisciplinary Sciences Wenzhou Institute, University of Chinese Academy of Sciences Wenzhou 325001 P. R. China; ^4^ Department of Chemistry Physics and Astronomy Stony Brook University Stony Brook NY 11794 USA

**Keywords:** cell cycle, landscape‐flux, nonequilibrium thermodynamics, single‐cell transcriptome

## Abstract

Recent developments in single‐cell sequencing technology enable the acquisition of entire transcriptome data. Understanding the underlying mechanism and identifying the driving force of transcriptional regulation governing cell function directly from these data remains challenging. This study reconstructs a continuous vector field of the cell cycle based on discrete single‐cell RNA velocity to quantify the single‐cell global nonequilibrium dynamic landscape‐flux. It reveals that large fluctuations disrupt the global landscape and genetic perturbations alter landscape‐flux, thus identifying key genes in maintaining cell cycle dynamics and predicting associated functional effects. Additionally, it quantifies the fundamental energy cost of the cell cycle initiation and unveils that sustaining the cell cycle requires curl flux and dissipation to maintain the oscillatory phase coherence. This study enables the inference of the cell cycle gene regulatory networks directly from the single‐cell transcriptomic data, including the feedback mechanisms and interaction intensity. This provides a golden opportunity to experimentally verify the landscape‐flux theory and also obtain its associated quantifications. It also offers a unique framework for combining the landscape‐flux theory and single‐cell high‐through sequencing experiments for understanding the underlying mechanisms of the cell cycle and can be extended to other nonequilibrium biological processes, such as differentiation development and disease pathogenesis.

## Graphical abstract

Single‐cell high‐throughput sequencing technology brings a lot of data, while statistical physics offers the potential for data analysis. A general framework is provided for combining the landscape‐flux theory and single‐cell high‐through sequencing data for understanding the underlying mechanisms of the cell cycle by learning the cell state force field based on discrete RNA velocity from the single‐cell transcriptome sequencing.

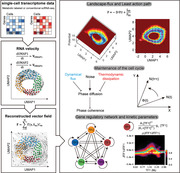

## 1 Introduction

The cell cycle is a complex process that controls cellular growth and division, playing a crucial role in the development and maintenance of living organisms.^[^ ^1^, ^2^ ^]^ An improved understanding of the regulation of the cell cycle is essential for comprehending various biological processes, including cellular differentiation, tissue development, and disease pathogenesis. Integrating statistical mechanics and single‐cell biology has provided novel opportunities to study the cell cycle and its regulation.^[^ ^3^ ^]^ Statistical mechanics offers a powerful framework for investigating complex systems, molecular interactions, and driving forces underlying cellular processes.^[^ ^4^ ^]^ Contrarily, single‐cell biology provides a method to explore cellular processes at the single‐cell level, which facilitates the comprehension of the heterogeneity of the cell populations and the mechanisms of cell differentiation.^[^ ^5^ ^]^ The precise study of the cell cycle is vital for understanding the life of a single cell, which is the fundamental unit of living systems. Conventionally, researchers explore the dynamics of the trajectories or perform local stability analysis around fixed points for the nonlinear system. However, this does not give a global picture or global stability, so people search further for landscapes to characterize the global stability of the system. However, the landscape alone can only quantify the dynamics of the equilibrium system with detailed balance (no net input to or output from), but it cannot describe the whole dynamics of the nonequilibrium system, an additional rotational flux as the driving force is necessary as we pointed out.^[^ ^6^ ^]^ The transitions between the gene expression states, which determine the driving forces, arise from a nonequilibrium effective potential landscape caused by the steady‐state probability and the rotational steady‐state probability flux between system states.^[^ ^7^ ^]^ The steady‐state probability flux measures the degree of the nonequilibrium and the irreversible behavior of a system, linking the dynamical and thermodynamic aspects of the system. Since flux originates from energy input and generates thermodynamic dissipation, it can act as a dynamic driving force.^[^ ^8^ ^]^ However, prior studies modeled the dynamics of cellular signaling networks based on the existing knowledge of gene regulatory networks, necessitating the estimation of several model parameters and network model simplification, deviating significantly from the real biological systems.^[^ ^9^, ^10^ ^]^ With advancements in single‐cell sequencing technology, complete transcriptomic data can be acquired. Several studies constructed the cell fate landscape from the state manifold of the single cell, which did not obtain the underlying mechanism of the cell signaling regulation.^[^ ^3^, ^11^, ^12^, ^13^, ^14^, ^15^, ^16^ ^]^ Consequently, studying the underlying mechanism via nonequilibrium dynamics and thermodynamics of transcriptional regulation of cell function directly from single‐cell transcriptomic data demands urgent attention.

Our work aims to contribute to the understanding of the cell cycle mechanism via nonequilibrium dynamics and thermodynamics by investigating the landscape‐flux quantified from the single‐cell transcriptomic data. Single‐cell RNA sequencing (scRNA‐seq) provides information on the gene expressions in the individual cells and enables us to analyze the gene expressions during the cell cycle. Through assessing the single‐cell RNA velocity, reconstructing vector fields, reducing dimensions, and mapping these patterns to lower‐dimensional spaces, we can visualize the landscape‐flux and understand the complex interactions between the different signaling processes that regulate the cell cycle, as well as the dynamic flux and thermodynamic dissipation that drive and maintain the cell cycle processes. We then performed in silico perturbations that enable us to identify key genes for cell cycle regulation and predict the effects of their genetic perturbations on the cell cycle dynamics and thermodynamics. Calculating the phase diffusion, coherence, and coherence time of the cell cycle oscillations, we quantified their relationships with dynamic flux and thermodynamic consumption. We found that noise mediates phase diffusion and reduces coherence and coherence time, resulting in shorter or even impossible cell cycle maintenance times. However, living cells continuously fight to maintain cell cycle oscillation by consuming energy, leading to a nonequilibrium process where entropy increases. We also found that the dynamic flux plays a role in maintaining the continuous cell cycle, which dynamically drives the cell continuously into the next cell cycle phase and promotes the coherent oscillation (cell cycle forward) to maintain the cell cycle oscillation. Lastly, we constructed the gene regulatory networks by identifying differentially‐expressed genes between different cell cycle phases and validated the key gene circuits behind cell cycle oscillation dynamics by calculating the Jacobian.

The cell cycle is crucial for cell division, governing cellular proliferation and development, which are the foundation of life. Our study employs a unique combination of experimental high‐throughput sequencing data and theoretical landscape‐flux approaches to understand the origin and driving forces of the cell cycle. The purpose of our research is to utilize high‐throughput data to experimentally verify the landscape and flux theory and provide its associated quantifications. Computational analysis identifies key genes and regulators critical for normal cellular function and compares them with the experimental data. Our work enhances our understanding of the biological functions of the cell cycle and has implications for maintaining normal cell function, which is crucial in disease prevention and human health. Moreover, the global principles from nonequilibrium dynamics and thermodynamics can be used to pin down the mechanisms in regulating cell cycle processes across organisms and underlying regulatory networks. In summary, our research represents an essential step forward toward comprehending the cell cycle and its regulation, potentially having a significant impact on the field of single‐cell biology and beyond.

## 2 Result

### 2.1 Quantifying Cell Cycle Nonequilibrium Landscape‐Flux from Single‐Cell Transcriptomics Data

Traditional systems biology studies have relied on a priori knowledge of biological regulatory networks to model and study cell dynamics; however, this approach is associated with several limitations. One major limitation lies in the simplification of networks, which may lead to an incomplete understanding of complex biological systems.^[^ ^17^ ^]^ Additionally, obtaining reliable results from these models can be challenging due to the estimation of a large number of parameters, such that they may not accurately reflect the real biological system.^[^ ^10^ ^]^ Although some studies have constructed a cell fate landscape from the state manifold of single cells, they are unable to obtain the underlying physical mechanism related to cell signaling regulation.^[^ ^13^, ^14^, ^15^, ^16^ ^]^ To overcome these limitations, we present a novel approach to studying the underlying mechanism of the cell cycle via nonequilibrium dynamics and thermodynamics by bypassing the prior known regulatory networks and quantifying the cell cycle regulatory dynamics directly from the experimental data collected by single‐cell high‐throughput sequencing.

**Figure 1:**
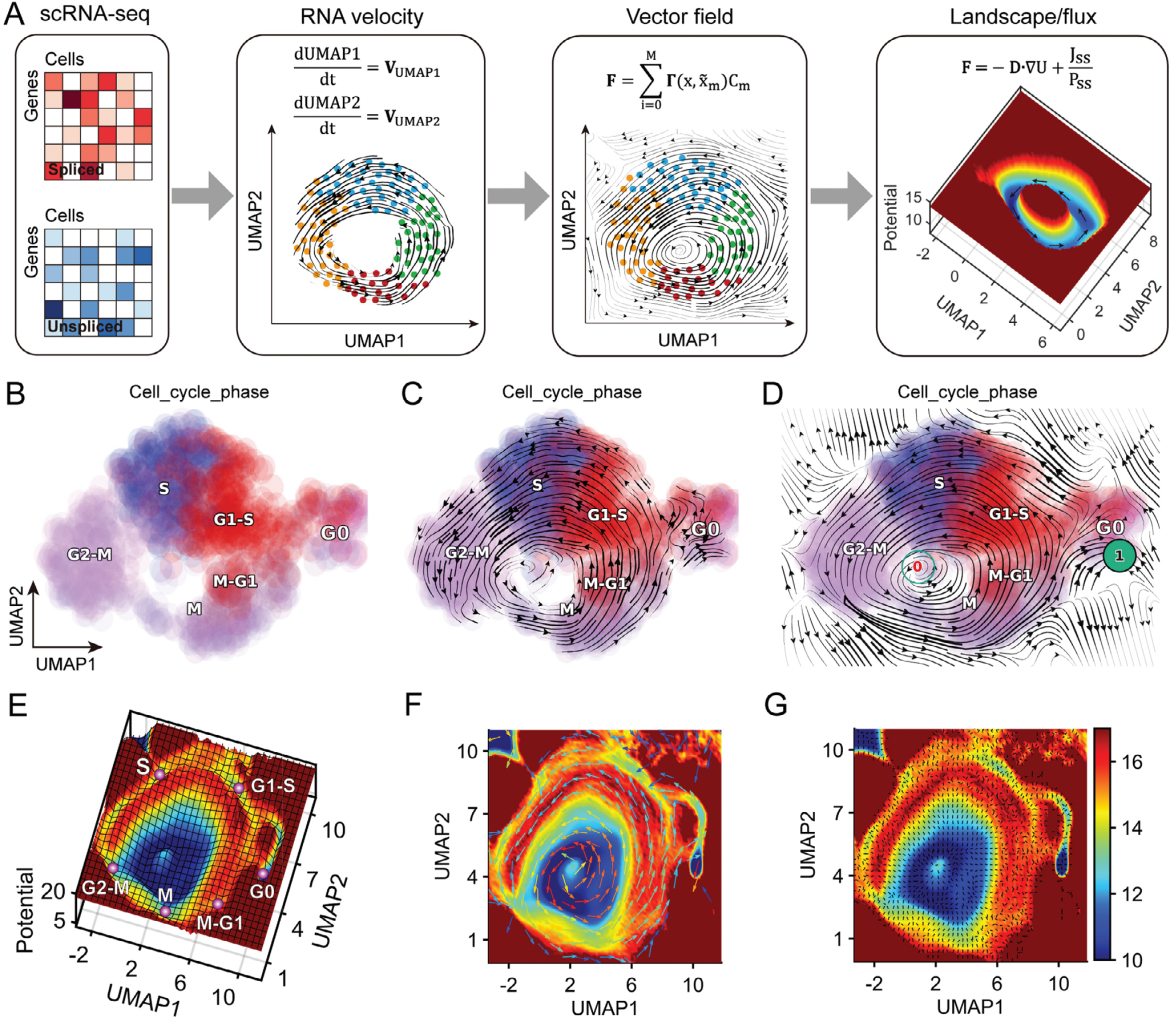
Workflow for analyzing cell cycle landscape‐flux from single‐cell transcriptomics data. A) Workflow of constructing cell cycle landscape‐flux by single‐cell transcriptomics data. B) Cell cycle phase clusters by UMAP of U2OS cells. C) RNA velocity of cell cycle dynamics of U2OS cells. D) Reconstructed vector field of cell cycle dynamics of U2OS cells. The red digit 0 represents the limit cycle attractor, reflecting cell cycle oscillations, and the black digit 1 represents the absorption fixed‐point attractor, reflecting the stationary phase of the cell at G0. E) Potential landscape of cell cycle dynamics in UMAP of U2OS cells. F) Curl flux (colored arrow) of cell cycle dynamics landscape in UMAP of U2OS cells. The color of the arrow presents the magnitude of flux, red corresponds to larger, and blue to smaller. G) The gradient force (black arrow) of cell cycle dynamics landscape in UMAP of U2OS cells.

We quantified the cell cycle dynamics landscape‐flux from the single‐cell transcriptomics data, following four primary steps (**Figure** **1**A). First, we downloaded and processed the cell cycle transcriptomics data, which provided information on gene expression patterns of individual cells over time for subsequent analysis. Second, we estimated RNA velocity from transcriptomics data. Third, we reconstructed the vector field of cell cycle dynamics from the RNA velocity information to visualize the movements of cells throughout the cell cycle. This captured the overall flow of cells through the cell cycle and provided us with a way to visualize the pseudo‐temporal trajectories of cells through the cell cycle. In the final step, the landscape‐flux of global cell cycle dynamics and thermodynamics was quantified. This method included UMAP‐based identification of cell cycle phase clusters, calculation of RNA velocity, reconstruction of cell cycle dynamics' vector field, computation of the cell cycle dynamic potential landscape, curl flux of the cell cycle dynamic landscape, and gradient force assessment of the cell cycle dynamic landscape.

To gain a deeper understanding of the underlying structure and flow of cells through the cell cycle, we performed a landscape‐flux analysis of the cell cycle dynamics using scRNA‐seq data from human bone osteosarcoma epithelial cells (U2OS cells),^[^ ^18^ ^]^ single‐cell 5‐ethynyl‐uridine‐labeled RNA sequencing (scEU‐seq) data from human retinal pigment epithelial‐1 cells (RPE1 cells)^[^ ^19^ ^]^ and scRNA‐seq data from human fibroblasts.^[^ ^20^ ^]^ We first identified cell cycle phase clusters in U2OS cells by applying the UMAP algorithm^[^ ^21^ ^]^ to generate a low‐dimensional representation of the cells (Figure 1B). Cells were then colored based on their cell cycle phase, allowing us to visualize the distribution of the cells in the cell cycle and to observe the heterogeneity of the cell cycle phases. The expressions of the transcription factors in the cells are different in different cell cycle phases, and the gene regulations are also different, so the cells have different cycle states (Figure S1, Supporting Information). RNA velocity^[^ ^22^, ^23^ ^]^ was then calculated for each cell to capture the direction and magnitude of changes in the gene expressions (Figure 1C), which facilitated the visualization of phase portraits and insight into cell cycle progression. The vector field of cell cycle dynamics was reconstructed using Dynamo,^[^ ^24^ ^]^ which allows us to reconstruct the function of the continuous and analytic velocity vector field from sparse, noisy single‐cell velocity measurements (Figure 1D). At the same time, we performed a differential geometry analysis of the cell cycle based on this vector field.^[^ ^25^ ^]^ Speed defines the length of the velocity vector in the vector field, that is, the magnitude of the velocity, which represents the magnitude of the force in the Langevin equation (Figure S2B, Supporting Information). Divergence characterizes the local flux exiting versus entering an infinitesimal region in the expression space – the “outgoingness”. The sources (sinks) often have strong positive (negative) divergence. The divergence in the cell cycle vector field represents the degree of convergence, reflecting the distribution of different cell cycle phases in the vector field (Figure S2C, Supporting Information). Curl characterizes the infinitesimal rotation of a cell state in the vector field, which is gradient‐free and represents the cell cycle state. The U2OS cell cycle velocity vector field is noise‐free, so the limit cycle of the cell cycle oscillation dynamics is small, and the curl is larger at the small limit cycle (Figure S2D, Supporting Information). The acceleration field reveals hotspots of cell states where the velocities change dramatically. When a cell leaves one cycle phase and enters another cycle phase, the acceleration is high. Acceleration represents an early driver of cell cycle regulation (Figure S2E, Supporting Information). The curvature field reveals hotspots where the velocity abruptly changes direction, such as regions around unstable fixed points where the gene expression changes from activation to repression or vice versa. Genes that contribute strongly to the curvature are drivers that control the cell fate. The curvature reflects the cell cycle phase bifurcation, which is larger in the G0 phase, indicating the switch between the oscillation‐limited cycle of the maintenance cycle and the G0 monostable state of the arrest cell cycle (Figure S2F, Supporting Information). Collectively, these results revealed hotspots of the cell states where velocities change dramatically, highlighting key transition points and driving forces of the cell cycle, including stability and instability. Using the reconstructed function, we also quantified the potential landscape U (U  =   − ln *P_ss_*) of the global cell cycle dynamics in UMAP, representing the steady state probability distribution *P*_ss_ of cells at different locations in different cell cycle phases with the shape of an irregular Mexican hat (Figure 1E). The Mexican hat‐shaped landscape features two ring valleys, and the small ring valley in the inner blue area is a virtual loop, and the real cell cycle oscillation trajectory corresponds to the outer ring valley.^[^ ^26^ ^]^ The emergence of such two different limit sets is a common phenomenon when performing dimensionality reduction with limit cycles to the UMAP plane, which is reminiscent of the Poincar´e‐Bendixson theorem in planar dynamics theory.^[^ ^27^ ^]^ The different phases of the cell cycle correspond to different basins of attractions (Purple balls) along the outer close oscillation ring valley path in the Mexcian landscape. Inside or outside the Mexican hat outer ring, the potential is higher and the probability of the system reaching these areas is low. The basin depth quantifies the duration of the stay in the corresponding phases and the local barrier between these phases or basins corresponds to the check point of the cell cycle.^[^ ^9^ ^]^ While the landscape attracts the system down to the cell cycle ring valley from the gradient part of the driving force, it is the curl flux from the rotational part of the driving force that drives the coherent oscillation of the cell cycle. Therefore, the Mexican hat shape landscape and the curl flux together guarantee the stability and robustness of cell cycle oscillation dynamics. This potential landscape also reflects the stability of different states in the cell cycle and highlights key transition points. Additionally, we quantified the curl flux and negative gradient force of the cell cycle nonequilibrium dynamics landscape in UMAP from the reconstructed function (Figure 1F,G). The curl flux indicates the amount of rotational flow in the cell cycle dynamics landscape that drives the cell cycle oscillations along the closed ring valley path, while the negative gradient force indicates the direction and magnitude of the forces stabilizing the cell cycle phase. If the driving force from the flux is greater than the force from the negative potential gradient, the cell will go across the G0 basin barrier into the G1‐S phase and the cell will enter the cycle phase. Otherwise, the cell will return to the G0 basin and be arrest in the G0 state. Biologically, this means that the nutrient supply is not large enough, or the DNA is damaged, as in the case of senescent cells.^[^ ^28^ ^]^

Similarly, we evaluated the RNA velocity from the scEU‐seq of the RPE1 cell cycle and scRNA‐seq of the human fibroblasts cell cycle, reconstructed the vector field for further differential geometric analysis (Figures S4 and S6, Supporting Information), and quantified the nonequilibrium landscape‐flux (Figures S3 and S5, Supporting Information). These findings verify the presence of the curl flux as another component of the driving force for the nonequilibrium dynamics in additional to the gradient of the cell cycle landscape by experimental single‐cell high throughput sequencing data, providing deeper insights into the mechanisms behind the stability and behavior of intrinsic nonequilibrium cell cycle progression that cannot be discerned by the landscape quantification alone. In fact, the cell cycle is one of the best examples of demonstrating the nonequilibrium dynamics being dictated by both the landscape and the flux. What's more, the creation of the cell cycle nonequilibrium landscape‐flux through single‐cell transcriptomics data presents a new and powerful tool for the study of cell cycle dynamics, allowing for insights into underlying mechanisms that drive cell cycle progression.

### 2.2 Noise Alters the Global Cell Cycle Dynamics and Thermodynamics

**Figure 2:**
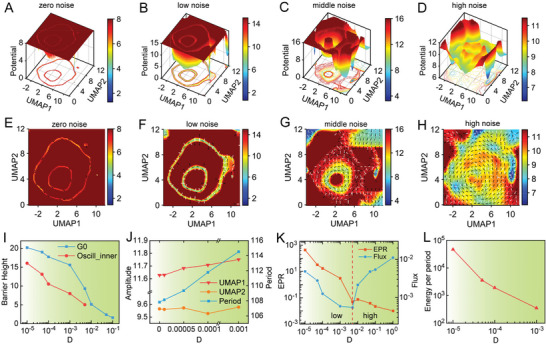
Cell cycle nonequilibrium landscape flux affected by noise intensity. A–D) Cell cycle global dynamics landscape of U2OS cells changes in UMAP when noise intensity is 0 A), 0.00005 B), 0.005 C), 0.05 D). E–H) Curl flux (black arrow) of cell cycle dynamics landscape in UMAP when noise intensity is 0 E), 0.00005 F), 0.005 G), 0.05 H). I) The change of Barrier Height of the G0 phase (blue) and oscillation center (red) in the landscape when the noise intensity is changed. J) The change of amplitude of UMAP1 and UMAP1 and the period of cell cycle oscillatory dynamics when the noise intensity is changed. K) The change of EPR and Flux of the cell cycle when the noise intensity is changed. L) The change of Energy per period of the cell cycle when the noise intensity is changed.

Variability in biomolecular noise or changes in molecular states can affect intracellular gene regulations and networks, which are crucial for the uncertain, multi‐destiny behavior of the system.^[^ ^29^, ^30^, ^31^ ^]^ To investigate the effects of noise intensity on the underlying mechanism of the cell cycle via nonequilibrium dynamics and thermodynamics, we performed simulations at varying noise intensities and analyzed the resulting global changes in the cell cycle dynamics and thermodynamics. First, we examined changes to the global cell cycle dynamic landscape and showed that as the noise intensity increases, the global dynamics landscape of the cell cycle becomes more complex and less predictable. Specifically, with zero noise, the cell cycle progresses very smoothly and predictably (**Figure** **2**A), while the ring valley of the cell cycle becomes wider as noise intensity increases (Figure 2B,C), whereas the ring valley is broken when the noise is high, showing the discrete attraction basins, at which time the cell cycle is broken and the cells arrest in the different phases (Figure 2D). Moreover, we found that the barrier height decreased in the G0 phase and oscillation center in the landscape as noise intensity increased (Figure 2I). The barrier height of the G0 phase is defined as the barrier from the G0 basin to the saddle point (between G0 and G1‐S), which quantifies how easily cells can switch from the quiescent G0 phase to the cyclic state. The barrier height of the oscillation center is defined as the potential difference between the minimum potential of the landscape and the local maximum inside the limit cycle, which characterizes the global stability of the oscillation system.^[^ ^7^ ^]^ We also investigated and found that the noise increases the cell cycle period, indicative of the rate at which the cell cycle oscillates and how fast it grows (Figure 2J; Figure S8, Supporting Information). The oscillation amplitude of UMAP1 increases with the noise intensity, resulting in a wider range of the oscillation limit cycle (Figure 2J; Figures S7 and S8, Supporting Information). Increased variability or noise effectively makes potential landscape basins shallower, thereby broadening the distribution of biological variables.^[^ ^32^ ^]^ By quantifying the trajectory autocorrelation function and the corresponding power spectrum density of the oscillating trajectory for different diffusion coefficients D, we can see that as the noise increases (the stability of the oscillation system decreases), the peak height of the power spectrum density decreases and the distribution of the power spectrum becomes more spread out (Figure S7, Supporting Information). This indicates that the peak height and the width of the power spectrum density can be a measure of the stability of the oscillation system. To be clear, when D is in the range of 0.01 to 0.1, the landscape is more realistic, with obvious cell cycle checkpoints, and it is consistent with the true level of experimental measurements.^[^ ^33^, ^34^ ^]^

Furthermore, we quantified the curl flux as a major component of the driving force of the cell cycle and the entropy production rate (EPR) as the measure of the thermodynamic cost association with the nonequilibrium curl flux of the cell cycle dynamic landscape to investigate how noise intensity affected the underlying mechanism of the cell cycle via nonequilibrium dynamics and thermodynamics. As shown in Figure 2E–H, increasing noise intensity resulted in a more widely distributed flux, with cells flowing between different phases less predictably. We observed that in the small noise range, the flux decreases as the noise increases, while the cell cycle is still oscillating (Figure 2K). Larger flux derived from the energy input or nutrient supply implies a larger cell cycle driving force and subsequently results in a shorter time to complete the cell cycle oscillation (the period is smaller). Indeed, the speed of the cell cycle is a hallmark of cancer. However, when the noise is very large, although the flux becomes larger with the increase of noise, the cell cycle is destroyed. At this point, the flux drive on the cell cycle can no longer resist the destruction of noise. In addition to global dynamics landscape alteration, noise intensity also affects the thermodynamics and energetics of the cell cycle. We found that the EPR of the cell cycle decreases with increasing noise intensity (Figure 2K). Furthermore, the energy per period of the cell cycle decreases with increasing noise intensity (Figure 2L), indicating a change in the energy requirements of the system. These results imply that the system requires more energy to maintain faster and more coherent cell cycle oscillations.

Taken together, these results demonstrate that noise intensity alters the underlying mechanism of the cell cycle via global nonequilibrium dynamics and thermodynamics, suggesting that the feedback of intracellular gene interactions plays an important role in maintaining the robustness and stability of the cell cycle in the face of environmental fluctuations. These findings have crucial implications for our understanding of the mechanisms that control cell cycle progression and may have important applications in the development of novel therapeutic strategies for the treatment of diseases associated with cell cycle dysregulation. These changes suggest that noise intensity may play a critical role in regulating the onset and termination of cell cycle phases.

### 2.3 In Silico Perturbation Analysis Cell Cycle Dynamics and Thermodynamics under Genetic Perturbation

**Figure 3:**
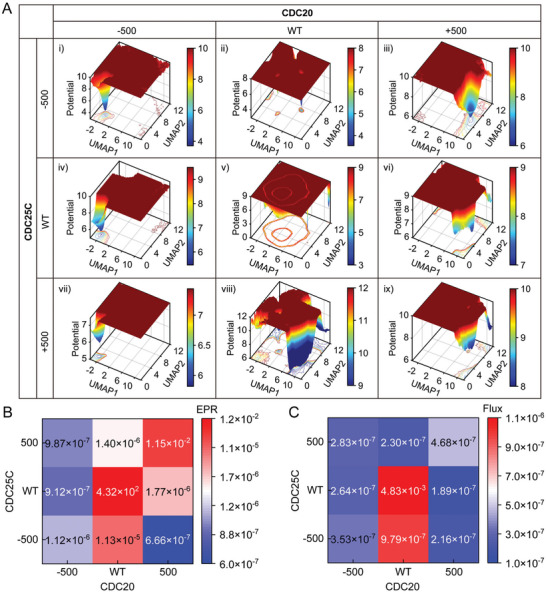
The genetic perturbation alters the cell cycle landscape/flux. A) Cell cycle global dynamics landscape of U2OS cells in UMAP with different genetic perturbations. Validation of in silico trajectory predictions. i) Suppression of both *CDC20* and *CDC25C*. ii) Suppression of *CDC25C* only. iii) Activation of *CDC20* and suppression of *CDC25C*. iv) Suppression of *CDC20* only. v) WT. vi) Activation of *CDC20* only. vii) Suppression of *CDC20* and activation of *CDC25C*. viii) Activation of *CDC25C* only. ix) Activation of both *CDC20* and *CDC25C*. B) EPR of cell cycle nonequilibrium thermodynamics with different genetic perturbations. C) The average Flux of cell cycle nonequilibrium dynamics with different genetic perturbations.

Genetic perturbation refers to the alteration of the function of a biological system by external or internal factors, such as environmental stimuli, drug inhibition, and gene knockdown.^[^ ^35^ ^]^ It is used to study how genes interact with each other and affect cellular processes and phenotypes.^[^ ^36^, ^37^, ^38^ ^]^ To investigate the effects of the genetic perturbations on the underlying mechanism of the cell cycle via nonequilibrium dynamics and thermodynamics, we performed in silico perturbations of different genes in a controlled and systematic manner. Key genes involved in cell cycle regulation, such as cyclin‐dependent kinases (CDKs), cyclins, and tumor suppressor genes, were perturbed in this study. Similar to Figure 1, we first estimated the RNA velocity of U2OS cells under different genetic backgrounds by in silico perturbation (Figure S9A, Supporting Information), and then reconstructed the different vector fields based on the perturbed RNA velocity (Figure S9B, Supporting Information). Finally, we quantified the global cell cycle dynamic landscapes under different genetic perturbations by adding noise to the dynamic simulation (**Figure** **3**A). We found that perturbing distinct genes resulted in specific changes in the cell cycle landscape and alterations in EPR and flux (Figure 3B,C).

Specifically, the negative perturbation of *CDC20* led to an increase in the barrier height of the G2‐M phase with a higher chance of arrest in this phase, while the positive perturbation of *CDC20* led to a deeper basin of the G1 phase with a higher chance of arrest in this phase (Figure 3A). As the experiment showed, *CDC20* plays a crucial role in cell division, being the target protein in the spindle checkpoint during mitosis.^[^ ^39^ ^]^ Pervious studies have found that *CDC20* expression is significantly increased in cutaneous squamous cell carcinoma tissues and cell lines,^[^ ^40^ ^]^ suggesting its role as a potential biomarker for diagnosis and treatment. We also found that the negative perturbation of *CDC25C* caused disruption of cell cycle oscillation and led to a deeper well of the G2‐M phase leading to possible arrest, albeit less obviously than the effect caused by perturbing *CDC20* (Figure 3A). Biologically, the downregulation of *CDC25C* induces cell cycle arrest in the G2/M phase in response to DNA damage via p53‐mediated signal transduction, and its abnormal expression is associated with cancer initiation, development, metastasis, occurrence, and poor prognosis.^[^ ^41^ ^]^ What's more, the negative perturbation of *CCNE2* led to an increase in the barrier height of the G2‐M phase with a higher chance of arrest in this phase, while the positive perturbation of *CCNE2* led to a deeper basin of the G1‐S phase with a higher chance of arrest in this phase (Figure S10, Supporting Information). While the negative perturbation of *CENPE* caused disruption of cell cycle oscillation and led to a deeper well of the G1‐S phase leading to possible arrest, the positive perturbation of *CENPE* caused the cell cycle to arrest in the G2‐M phase. Researchers observed disorientation of cell division in the cells of patients with *CENPE* mutations in microcephaly,^[^ ^42^ ^]^ and our genetic perturbation findings provide additional insight into how microcephaly is caused by centrosome defects. Similarly, genetic perturbations in RPE1 cells are manifested that the negative perturbation of *KPNA2* led to a higher barrier height of the G2‐M phase, whereas the positive perturbation of *CDC20* led to a higher barrier height of the M phase (Figure S11A, Supporting Information). This finding aligns with experimental evidence: *KPNA2* knockdown downregulated *CCNB2* and *CDK1*, inhibited cell proliferation, and induced cell cycle arrest in the G2‐M phase.^[^ ^43^ ^]^ *CCNE2* is a regulatory subunit of cyclin‐dependent kinase 2 (Cdk2) and is thought to control the transition of quiescent cells into the cell cycle.^[^ ^44^ ^]^ Our genetic perturbations also confirm the cell cycle initiation function of *CCNE2*, as reflected in the deepening of the G0 well under negative perturbation of *CCNE2*, and the disappearance of the G0 basin with the appearance of other periodic phases following positive perturbation of *CCNE2* (Figure S11A, Supporting Information).

Figure 3B shows the EPR of cell cycle nonequilibrium thermodynamics with different genetic perturbations. We observed that different genetic perturbations can cause significant changes in EPR, with some resulting in increased energy consumption and others in decreased consumption. However, the EPR is lower under genetic perturbations than in WT, strong perturbations disrupt cell oscillatory dynamics (Figures 3B; Figure S11B, Supporting Information). Thus, maintaining continuous cell oscillation requires greater energetic costs than arresting in different periodic phases. We also quantified the average flux of cell cycle nonequilibrium dynamics with various genetic perturbations and found that WT cells exhibit the largest curl flux, where the flux as a driving force of the cell cycle is greater than the negative gradient force, and the flux drives the cell cycle to continue moving forward to maintain the coherent oscillation (Figures 3C; Figure S11C, Supporting Information). In contrast, specific genetic perturbations result in a flux less than the negative gradient force, insufficient to drive the cell cycle forward, and causing cells to arrest at certain cell cycle phases.

Overall, the results demonstrate the efficacy of using the reconstructed vector field based on RNA velocity derived from single‐cell omics data of the cell cycle to predict how genetic perturbations impact cell cycle behavior. This methodology has the potential for identifying the critical regulatory genes involved in maintaining cell cycle coherent oscillation, such as *CDC25C*, *CCNE2*, *KPNA2*, etc., which may be the new targets in cancer.

### 2.4 The Underlying Mechanism of Cell Cycle Initiation and Termination via Nonequilibrium Dynamics and Thermodynamics

The ability to drive cellular state transitions has gained attention as a promising strategy for disease modeling.^[^ ^45^ ^]^ The quantified Waddington landscape provides a way of describing and revealing biological pathways of developmental processes.^[^ ^46^ ^]^ A previous study confirmed that gradient and curl forces control the dynamics of these biological paths on the landscape, rather than following the steepest descent path expected.^[^ ^17^ ^]^ To investigate the cell cycle progression and transitions between different phases, we computed the Least Action Paths (LAPs) and Mean First Passage Time (MFPT) between different cell cycle phases. LAPs are optimal phase space trajectories minimizing the action's cost of moving from one state to another, while MFPT is the average transition time between states. Specifically, the optimal path between any two cell states (e.g., the fixed points of G0 phase and G1/S phase) is searched by changing the continuous path connecting the source and target states while minimizing its effect and updating the associated transition time.^[^ ^9^ ^]^ The resulting LAP has the highest transition probability and is correlated with a specific transition time.

**Figure 4:**
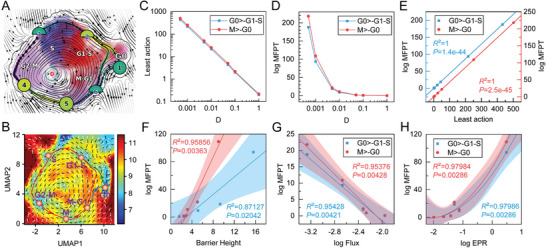
LAPs and MFPT in cell cycle initiation and termination. A) LAPs between different cell cycle phases in the velocity vector field of U2OS cells. The color of digits in each node reflects the type of fixed point: red, emitting fixed point; black, absorbing fixed point. The color of the numbered nodes corresponds to the confidence of the fixed points. The color of the dots along the paths corresponds to the direction. B) LAPs between different cell cycle phases in the 2D landscape of U2OS cells. The black arrows present the curl flux and the white arrows present the gradient force in the landscape. C) The least action of G0 phase to G1‐S phase and M phase to G0 phase versus diffusion coefficient *D*. D) The logarithm of MFPT of G0 phase to G1‐S phase and M phase to G0 phase versus diffusion coefficient *D*. E) The correlation between the least action and the logarithm of MFPT when diffusion coefficients (fluctuations) are changed. F) The correlation between barrier height and the logarithm of MFPT when diffusion coefficients are changed. The line is a fitting correlation line, and the shaded part is the 95% fitting confidence interval. G) The correlation between flux and the logarithm of MFPT when diffusion coefficients are changed. H) The correlation between the logarithm of EPR and the logarithm of MFPT when diffusion coefficients are changed.

We first map the LAPs between different cell cycle phases in the velocity vector field, where the nodes along the pseudo‐temporal paths represent the discrete points, with color indicating LAP transition pseudo‐time and arrows depicting pseudo‐temporal path direction, and the black curves connecting different phases representing LAPs (**Figure** **4**A; Figure S12D,F, Supporting Information). We also calculated and illustrated the LAPs on the landscape, which reveals the optimal path (least action) that cells take to overcome potential barriers during the cell cycle (Figure 4B; Figure S12E,G, Supporting Information). Green curves connect G0 to G1‐S and blue curves connect M to G0, representing the cell cycle's initiation and termination, respectively. The cell cycle path (red curves) is nonuniform and sandwiched between flux (black arrows) and negative potential gradient (white arrows), demonstrating that progression arises from both curl flux and negative potential gradient force. To enter the cycle from the G0 basin to the G1‐S basin, the cells must overcome a barrier on the path (saddle point on the landscape) located between the G0 and G1‐S basins, where the flux acts as a driving force.

In addition to calculating the LAPs, we also quantified the pseudo‐time of the cell cycle initiation and termination (Figure 4C–E). We indicated that noise reduces the least actions of cell cycle initiation and termination, resulting in a decrease in MFPT, suggesting that strong fluctuations make it easier for cells to enter the cycle, but also easier to terminate the cell cycle when the cycle coherence is weakened. Furthermore, we found that the logarithm of MFPT is directly proportional to the barrier height on the landscape, i.e., the higher the barrier, the more stable the cell state, and the more difficult it is for the cell to leave this cycle phase (the larger the least action and the longer the escape time) (Figure 4F; Figure S12A, Supporting Information). The barrier heights between the attraction basins correlate with the escape time, which characterizes the stability of the cell cycle phase. Additionally, the logarithm of MFPT is negatively correlated with the logarithm of nonequilibrium dynamic flux, suggesting that the larger the flux, the stronger the driving force, and the easier it is for cells to escape a given steady state (Figure 4G). However, thermodynamic energy cost‐wise, the logarithm of MFPT is positively correlated with the logarithm of EPR (Figure 4H). The longer the cell takes to escape, the more energy it consumes. To overcome this barrier, cells must expend a certain amount of energy, known as cell cycle initiation energy.^[^ ^47^ ^]^ In summary, these results provide a quantitative assessment of the initial energy required for the progression of the cell cycle (e.g., nutrient supply capacity and metabolic activity^[^ ^48^ ^]^) and provide graphical, physical, and quantitative explanations for the checkpoint mechanisms of the cell cycle.

### 2.5 Maintenance of the Cell Cycle Requires Energy‐Dissipation and Flux to Enhance the Coherence of the Oscillation Phase

**Figure 5:**
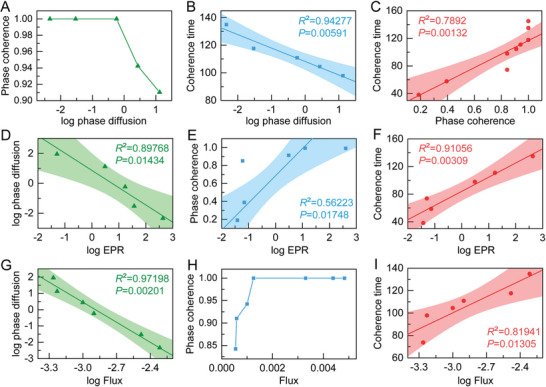
Relationship between phase diffusion, phase coherence, coherence time, and EPR and flux. A) Phase coherence versus the logarithm of phase diffusion. B) The correlation between coherence time and the logarithm of phase diffusion when diffusion coefficients (fluctuations) are changed. The line is a fitting correlation line, and the shaded part is the 95% fitting confidence interval. C) The correlation between coherence time and phase coherence when diffusion coefficients are changed. D) The correlation between the logarithm of phase diffusion and the logarithm of EPR when diffusion coefficients are changed. E) The correlation between phase coherence and the logarithm of EPR when diffusion coefficients are changed. F) The correlation between phase coherence and the logarithm of EPR when diffusion coefficients are changed. G) The correlation between the logarithm of phase diffusion and the logarithm of Flux when diffusion coefficients are changed. H) The change of phase coherence when Flux is changed. I) The correlation between coherence time and the logarithm of Flux when diffusion coefficients are changed.

Biological systems need to function accurately in the presence of strong noise, and various regulatory mechanisms have evolved to suppress the fluctuation and environmental effects in processing vital life processes such as cell cycle and development.^[^ ^49^ ^]^ In a noisy environment, cell cycle oscillations can be highly inaccurate due to phase fluctuation, the phase of oscillation follows diffusive dynamics.^[^ ^50^, ^51^ ^]^ A previous study found that phase coherence is a property of waves that describes how well‐aligned the phases of the waves are. When waves are perfectly coherent, their peaks and troughs align precisely, creating a stable and predictable pattern.^[^ ^7^, ^52^ ^]^ We quantified the relationship between noise intensity and cell cycle oscillation properties (phase diffusion, phase coherence, and coherent time) and found that noise fluctuations increase phase diffusion, decrease phase coherence, and make the coherence time (cell cycle maintenance time) to become lower (Figure S13, Supporting Information). In the system of cell cycle regulation, phase diffusion refers to the random fluctuation of the phase of oscillating waves. After certain times, the oscillations can lose the memory or track of the previous dynamics. This makes it harder to maintain the periodicity. The phase coherence is important for maintaining accurate and reliable oscillations in noisy environments. Coherent time refers to the duration over which a system exhibits coherence, or the ability to maintain a stable and predictable pattern.^[^ ^53^ ^]^ In other words, it is the length of time during which oscillating waves in the cell cycle system remain perfectly aligned. There are significant correlations between these factors: increasing phase diffusion leads to a corresponding decrease in phase coherence, and phase diffusion can reduce the coherent time of the cell cycle system, especially in large fluctuation systems (**Figure** **5**A,B). Decreased phase coherence leads to decreased coherent time because less well‐aligned waves become less stable and predictable over time (Figure 5C).

To identify the nonequilibrium mechanism of the cell maintenance cycle progression, we further investigated the effects of dynamic flux and thermodynamic dissipation on the characteristic features of cell cycle oscillations. Our results indicate that the logarithm of phase diffusion is negatively correlated with the logarithm of EPR, while phase coherence and coherent time are positively correlated with the logarithm of EPR (Figure 5D–F). This suggests that thermodynamic dissipation plays an essential role in regulating the overall dynamics of the cell cycle oscillation. As cells enter the cycle, metabolic processes generate ATP and other energy molecules to provide thermodynamic energy dissipation to maintain the cell cycle oscillations.^[^ ^54^ ^]^ This added energy can help counteract the effects of phase diffusion, maintaining coherence, and resulting in more reliable and effective signaling between different cell cycle phases.^[^ ^50^ ^]^ We also found that changes in the logarithm of phase diffusion were negatively correlated with changes in the logarithm of dynamic flux (Figure 5G). Furthermore, we observed that higher flux levels were associated with increased phase coherence and coherence time (Figure 5H,I). These findings highlight the critical role of nonequilibrium dynamic flux force in increasing the stability of the cell cycle flow by enhancing phase coherence and coherent time.

Overall, these results suggest that there is a cost‐performance trade‐off for noisy biochemical oscillations, where increasing phase coherence comes at a higher thermodynamic cost, which aligns with the principles of thermodynamic uncertainty relationship (TUR). TUR establishes a trade‐off between the dissipation of energy and the precision of observed quantities. In the context of cell cycle oscillating systems, maintaining coherence in the oscillation phases may require the dissipation of energy to counteract the natural tendency of the system to relax toward equilibrium. The coherent oscillation necessitates energy input to sustain the oscillatory behavior and counteract the dissipative processes that tend to disrupt coherence. the TUR can guide the understanding of how energy allocation influences the coherence and precision of oscillation phases. While the energy cost of an oscillating system can counteract the effects of phase diffusion and maintain coherence over longer periods of time, continuous energy input is required to sustain the system's oscillations, which can be energetically costly in certain circumstances. The energy cost of cell cycle progression is essential for ensuring that cells divide in a controlled and regulated manner, but excessive energy cost or disruptions in energy metabolism can result in cell cycle defects and potentially serious health problems such as cancer. Therefore, regulating energy metabolism and maintaining cell cycle oscillations are critical for preserving the health and proper function of cells and tissues.

### 2.6 Cell Cycle Gene Regulatory Network Inference from Single‐Cell Transcriptomics Data

**Figure 6:**
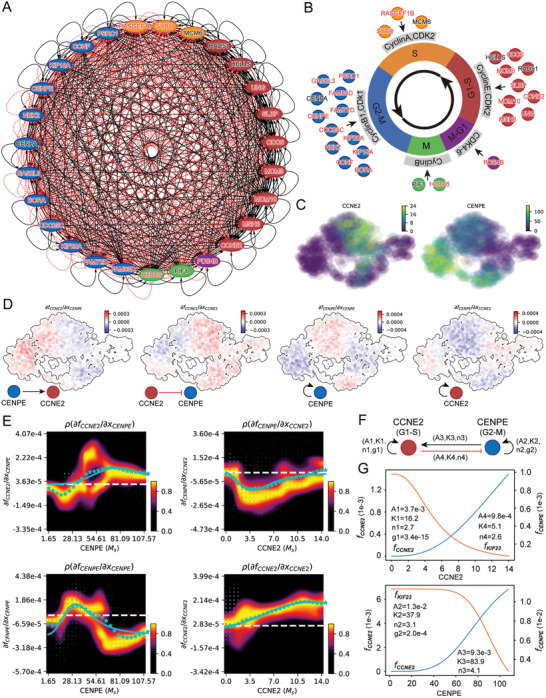
Inference of gene regulatory networks for the cell cycle by single‐cell transcriptomics data. A) The interaction network of genes of each cell cycle phase of U2OS cells. All differentially expressed genes in each cell cycle phase are used to construct the network; nodes (genes) with more than one edge are shown. Node colors represent different cycle phases (red: G1‐S, yellow: S, blue: G2‐M, green: M, purple: M‐G1), the black arrows represent the activation and the red arrows represent the inhibition. Genes with black representing TF and red representing non‐TFs. B) The diagram for the cell cycle model with key genes of each cell cycle phase. C) *CCNE2* has high expression in the G1‐S phase and *CENPE* has high expression in the G2‐M phase. D) Molecular mechanisms underlying the maintenance of the cell cycle. i) *CENPE* activates *CCNE2*. ii) Repression of *CENPE* by *CCNE2*. iii) Self‐activation of *CENPE*. iv) Self‐activation of *CCNE2*. E) Fitting the function of Jacobian versus gene expression with derivatives of a simplistic inhibitory or activation Hill equation. The White dashed line corresponds to the zero Jacobian value. The blue stars at each x‐axis grid point correspond to the weighted mean of the Jacobian values for that point. The blue solid lines are the resultant fittings for the Jacobian. F) Schematic summarizing the interactions involving *CCNE2* and *CENPE*. G) The velocity kinetic curves over gene expression changes of the corresponding fitted Hill equations of (E).

Gene expression does not appear in isolation, which is influenced by a complex interplay of regulatory interactions with other genes and small molecules. The discovery of these regulatory interactions is the aim of gene regulatory network (GRN) inference methods.^[^ ^55^, ^56^, ^57^, ^58^, ^59^, ^60^, ^61^ ^]^ The use of gene regulatory network modeling to analyze biological dynamic processes is an interdisciplinary field that has been widely studied in systems biology. To decode the regulatory mechanism of cell cycle phases, we first identified 27 and 16 differentially expressed genes of the U2OS cell and RPE1 cell, separately (Tables S1 and S2, Supporting Information), which were used to construct the genetic network that underlies each cell cycle stage. The analytic results demonstrate that the enriched expression of cycle phase‐specific genes regulates and maintains cells into different cycle stages via corresponding niche components (**Figure** **6**A; Figure S16A, Supporting Information). The nodes in the network represent genes colored according to their associated cell cycle phase. We also identified key genes for each cell cycle phase and constructed a wiring diagram for the cell cycle network model with these key genes (Figure 6B; Figure S16B, Supporting Information). The GRN identifies key genes for each cell cycle phase known to play important roles in different cell cycle phases and biological processes such as DNA replication and mitosis. For instance, *CDC6*, *MCM3*, and *CCNE2* are key regulators of G1/S transition and cell cycle initiation that regulate multiple GRNs (CyclinE and CDK2) in the G1‐S phases.^[^ ^44^, ^62^ ^]^ While the S phase is regulated by *MCM6*, *AURKB*, and *CDK1*, which are related to DNA replication and phosphorylate several key proteins to control the transition from the G1 to S phase of the cell cycle.^[^ ^63^, ^64^, ^65^ ^]^ Other important genes identified include *CDC25C*, *CENPE*, and *KIF23*, which regulate the G2/M checkpoint by affecting mitotic cell cycle.^[^ ^66^, ^67^, ^68^ ^]^ Additionally, the GRN reveals that the M phase is regulated by *CDC20*,^[^ ^39^ ^]^ *PIF1*,^[^ ^69^ ^]^ and *TPX2*,^[^ ^70^ ^]^ *PDE4B*,^[^ ^71^ ^]^ and *KIF20A*,^[^ ^72^ ^]^ highlighting the crucial role of these factors in controlling the M‐G1 phase.

Although there are many methods for inferring gene regulatory networks, most of them cannot obtain the strength of regulation, and only a few tools can do it, including Dynamo,^[^ ^24^ ^]^ spliceJAC,^[^ ^57^ ^]^ etc. In our study, we used Dynamo, based on the differential geometry analysis of the reconstructed cell cycle vector field from the single‐cell transcriptomics data, we reveal the quantitative information about gene regulation and further uncover the molecular mechanism underlying the maintenance of the cell cycle and the interactions between the key genes. Taking U2OS cells as an example, we demonstrate via our inferred regulatory network that *CCNE2* and *CENPE* regulate the G1‐S and G2‐M phases, respectively, showing peak expression in their respective regulatory cycle phases (Figure 6C). Jacobian analysis of vector fields can help us study cellular state‐dependent gene interactions, with positive and negative values of the Jacobian matrix reflecting activation and inhibition regulation, respectively.^[^ ^24^ ^]^ Across all cells, Jacobian analyses demonstrate the activation of *CCNE2* by *CENPE*, repression of *CENPE* by *CCNE2*, self‐activation of *CENPE*, and self‐activation of *CCNE2* (Figure 6D). These results collectively suggest that the interlinked positive and negative feedback loops between *CCNE2*, which regulates the G1‐S checkpoint, and *CENPE*, which regulates the G2‐M checkpoint, underlies the dynamics of cell cycle oscillations, revealing the critical role of two transcription factors in maintaining cell cycle progression (Figure 6F). Although only one negative feedback loop is required for biological oscillation, we found that *CCNE2* and *CENPE* have additional self‐activating positive feedback loops that promote modularity of cell cycle frequency while essentially unchanged amplitude (Figure 2J; Figure S8, Supporting Information). Previous computational studies have shown that it is often difficult to adjust the frequency of a negative feedback oscillator without affecting the amplitude, whereas, with positive plus negative feedback, a widely adjustable frequency and a near‐constant amplitude can be achieved.^[^ ^73^ ^]^ This also reveals that keeping the cell cycle more robust and easier to evolve requires a lot of positive and negative feedback coupling. To further quantitatively understand the feedback regulation mechanism between regulators, we first plotted distributions of the four Jacobian elements versus the expression of each gene (Figure S14A–D, Supporting Information). Then, we estimated kinetic parameters with derivatives of a simplistic inhibitory or activation hill equation by fitting the Jacobian versus expression curve in the response heatmap (Figure 6E). Finally, we theoretically and numerically simulate the velocity dynamics curve of the hill equation as a function of gene expression (Figure 6G). The GRN inferred above not only indicates the presence or absence of the interaction between genes but does provides quantitative information on positive and negative regulation relationships. Similarly, based on the REP1 cell cycle regulation network inferred from scEU‐seq data, we further revealed the feedback mechanism between *CCNE1* (a G1‐S phase regulator) and *KIF23* (a G2‐M phase regulator) and quantified the intensity of feedback between each other (Figures S14E–H and S16, Supporting Information).

During the cell cycle, dynamically evolving gene regulatory networks (GRNs) are sculpted by an array of cell‐cycle phase‐specific transcription factors. A deep understanding of the dynamic changes in network topology across different cell cycle stages is essential for a holistic view of the cell cycle. To examine the dynamic changes in network topology across different cell cycle stages, We utilize the spliceJAC^[^ ^57^ ^]^ package to infer and illustrate the cell cycle phase‐specific gene regulatory networks related to U2OS and RPE1 cell cycle by quantifying the Jacobian of gene‐gene interaction matrices for different cell cycle phases (Figures S15A and S17A, Supporting Information). We found that the *KRT34* gene has the highest betweenness centrality in the GRN of the G1/S phase of the U2OS cell cycle and *IL13RA2* has the strongest inhibition of *FAM11B* (Figure S15B). During the S‐cycle phase, the *PTP4A3* gene has the highest betweenness centrality, and *A2M* has the strongest inhibition of *FAM11B*. In the G2‐M stage, the *HAS2* gene has the highest betweenness centrality, and *AKR1C3* has the strongest inhibition of *EGR1*. While at the M and M‐G1 stages, the *H2BC4* gene has the highest betweenness centrality, and *PTP4A3* has the strongest inhibition of *FAM111B*. Our analysis revealed that gene regulatory networks exhibit a variety of phase specificity: the cell cycle‐phase specificity in the expression level of genes, the interaction intensity between genes, and the type of interactions between genes (Figures S15B and S17B, Supporting Information). By inferring the feedback mechanism between different cell cycle phase regulators, we confirmed that the cell cycle regulatory network is composed of motifs coupled with many negative feedback loops, which together constitute a topology that maintains periodic oscillation. These analyses not only infer the regulatory network of cell cycle function from single‐cell omics data, so as to find out the topological basis of cell function but also further quantify the interaction strength between the regulatory factors, which can provide newly quantitative and accurate guidance for the on‐demand design of functional elements in synthetic biology.^[^ ^74^ ^]^

## 3 Discussion

Spectacular advances in experimentation during the last decade, for example, single‐cell dynamics in microscopy and in high‐throughput data acquisition, have yielded an unprecedented wealth of data on cell dynamics, genetic regulation, and organismal development. These experimental data have motivated the development and refinement of concepts and tools to dissect the physical mechanisms underlying biological processes. An appropriate analytical framework is needed to use these data to study the underlying mechanisms through the nonequilibrium dynamics and thermodynamic behavior of cells. Our study utilizes high‐throughput sequencing experimental data to verify the landscape and flux theory and provides its associated quantifications. The cell cycle is a complex process that is tightly regulated to ensure proper cell growth, differentiation, and proliferation. Dysregulation of the cell cycle can lead to various diseases, including cancer. Therefore, understanding the fundamental mechanisms that govern the cell cycle is critical for advancing our knowledge of cellular physiology and developing therapeutic strategies for treating diseases. In this study, we utilized an integrative approach combining single‐cell transcriptomics with nonequilibrium statistical physics to better understand the complex regulatory networks and analyze the molecular events involved in the cell cycle at the single‐cell level to fill this unmet gap. This process includes processing the upstream dimension reduction clustering of single‐cell transcriptome data of the cell cycle, estimation of RNA velocity, reconstruction of cell cycle vector fields, and quantification of nonequilibrium dynamic and thermodynamic landscapes‐flux.

Conventionally, researchers have focused on examining the dynamics of trajectories or analyzing the local stability of nonlinear dynamical systems. However, these approaches often offer only a local view of the system's stability and behavior. To obtain a more comprehensive understanding of the overall stability and behavior, people have turned to the concept of landscape, which can describe the global stability of the system. However, it has become apparent that relying solely on the landscape for the dynamics is true only for equilibrium systems with detailed balance (no net input to or output from the system) but is insufficient for capturing the entire dynamics for the nonequilibrium systems.^[^ ^7^, ^8^, ^9^, ^17^, ^46^, ^75^, ^76^ ^]^ Our findings in this study of the cell cycle illustrate this point well, as its complex behavior requires the presence of an additional rotational flux. While the landscape contributes to attracting the system into the close oscillation ring valley, it is the flux that drives the coherent oscillation along the cycle path. Notably, our previous studies have experimentally confirmed and quantified probability flux as the driving force for nonequilibrium dynamics and thermodynamics in biochemical systems.^[^ ^77^, ^78^ ^]^ Not only extending the previous steady‐state theory to the case of cell cycle dynamics, our study also provides a novel framework for combining the landscape‐flux theory and single‐cell high‐throughput sequencing data for understanding the underlying mechanisms of the biological important processes for the cell fate decision‐making and the oscillations such as the cell cycle. On the other hand, this manuscript utilizes single‐cell transcriptomics data and performs in silico perturbations to identify certain new key genes for cell cycle regulation, which cannot be found in the previous modeling studies. By combining both concepts, one can gain deeper insights into the mechanisms behind the stability and behavior of complex systems.

One of the key findings of our research is the significant impact of biological noise on the underlying mechanism of the cell cycle via nonequilibrium dynamics and thermodynamics. In the small noise range, when the cell cycle oscillation is not disrupted, the noise increases the cell cycle amplitude and period, accompanied by a decrease in the curl flux and EPR. Larger flux means more energy input and larger cell cycle driving force and therefore leads to less time to complete the cell cycle oscillation (the period is smaller). Increased noise effectively makes potential landscape basins shallower, thereby broadening the distribution of biological variables. After the noise increases to a certain extent, the potential energy landscape shows completely different basins, the cell cycle is broken, and the cells arrest in different cycle stages (attractor‐basins). At this time, as the noise increases, although the curl flux increases, it appears that the dominant role of the noise masks the energy supply which is not enough to maintain the coherent oscillation. Notably, these studies have experimentally confirmed that the ATP level tends to peak near cell division and the cell cycles with the smaller ATP fluctuations correlate with the higher growth rates.^[^ ^54^ ^]^ Our study also demonstrated that the key genetic perturbations can significantly alter the underlying mechanism of the cell cycle via nonequilibrium dynamics and thermodynamics. Specifically, perturbations of the cell cycle gene related to cell cycle initiation, DNA replication, or mitosis all lead to landscape‐flux changes that are consistent with the biological effects that have been experimentally validated.^[^ ^39^, ^41^, ^43^, ^44^ ^]^ These highlight the importance of the genetic perturbations in the cell cycle and can be used to predict the pathological effects of certain genetic perturbations, thus providing a framework for understanding the molecular basis of the genetic diseases and diseases that influence the cell cycle. By identifying the bottlenecks and rate‐limiting steps in the cell cycle, researchers may be able to develop targeted therapies that can disrupt the cell cycle and selectively kill cancer cells.^[^ ^79^ ^]^ Therefore, future research could build on these findings to develop more accurate models of the cell cycle and other biological processes and to explore the potential impact of genetic and environmental perturbations on these systems.

We also studied the underlying mechanism via nonequilibrium dynamics and thermodynamics of the cell cycle initiation and termination by counting MFPTs and LAPs between different cell cycle phases. In general, MFPT is defined as the average transition time of a system switching from one state to another, which can be measured experimentally and accurately.^[^ ^80^ ^]^ The LAP we calculated based on single‐cell data is the pseudo‐time trajectory, and the MFPT is also pseudo‐time, not necessarily the true gene expression time. The positive correlation between barrier height and MFPT more intuitively shows the reasons for the difficulties of cell initiation and termination from the 3D landscape, and it is worth noting that our previous works can experimentally quantify the landscape and barrier heigh.^[^ ^80^, ^81^ ^]^ The analysis of EPR and MFPT reveals the existence of basal energy supply in the cell cycle, as well as the initial energy dissipation for cell cycle initiation,^[^ ^47^ ^]^ which approximates the product of EPR and MFPT from G0 phase to G1‐S phase. We also found that the cell cycle is a highly energy‐dissipative process, and the maintenance of the cell cycle requires thermodynamic energy‐dissipation and dynamic flux to enhance the coherence of the oscillation phase against stochastic fluctuations. From a thermodynamic point of view, living systems are open systems in a nonequilibrium state, requiring material input and energy consumption to maintain their steady‐state and order.^[^ ^82^, ^83^ ^]^ The thermodynamic uncertainty relationship is a result of nonequilibrium statistical mechanics that relates the dissipation of energy or entropy production to the precision of a thermodynamic observable. In systems far from equilibrium, there is a trade‐off between the dissipation of energy (or entropy production) and the fluctuations in the observed quantities. The TUR establishes a lower bound on the fluctuations of a thermodynamic observable based on the rate of entropy production or energy dissipation in the system. The cell cycle is naturally a living system, G0 phase is a nondividing state of the cell cycle, in which the cell is metabolically active but not actively dividing. To switch from the G0 phase to the G1/S phase, the cell requires a certain amount of energy to initiate the complex set of biochemical reactions that drive cell division. Once the cell has entered the G1/S phase, it must maintain a complex system of oscillations and feedback loops to progress through the rest of the cell cycle. This requires additional energy input to maintain the oscillations of key cell cycle regulators such as cyclins and cyclin‐dependent kinases (CDKs). Currently, certain experiments can quantify the energy flow by measuring the rate of oxygen consumption or heat production or the fluxes of metabolites including the production and consumption of ATP.^[^ ^84^ ^]^

In systems biology, the creation of core network regulation to analyze biological processes is an important and popular topic. In general, there are two ways to build a biological regulatory network: one is to create a small network based on the existing knowledge and database and use the simulator to improve the network, but it is inefficient and cannot be used to build a new network model.^[^ ^85^, ^86^ ^]^ The second is to use bioinformatics technology, especially high‐throughput sequencing data, to infer genes and gene correlations, but ignore the actual biological modulation relationship.^[^ ^87^ ^]^ Of course, there are different methodological models for inferring gene regulatory networks from single‐cell data, the different models used in the inference can lead to different inference outcomes.^[^ ^88^ ^]^ Ultimately, which method is better should be tested and determined by the experiments. To balance the advantages and disadvantages of these two approaches, we infer the transcriptional regulatory networks based on differentially expressed genes of each cycle phase from single‐cell transcriptomics data. The regulatory direction between genes (activation/inhibition) can be inferred by Jacobian analyses of vector fields and the hill function of the core network can be simulated by fitting Jacobian in the gene expression space and response heatmap. We reveal that cell cycle regulations involve multiple positive and negative feedback loops between these regulators, which help to adjust the frequency of the oscillation without significantly affecting the amplitude, enabling widely adjustable frequencies and near‐constant amplitudes and maintaining the balance between the activation and inhibition of the gene expressions, permitting for proper progression through different cell cycle phases. Our results identify that some dynamic biological processes can be accurately mapped by constructing regulatory network models based on high‐throughput sequencing data. These gene regulatory networks, inferred from the single‐cell sequencing data, are found to be highly consistent with the experimental results.^[^ ^39^, ^44^, ^62^, ^63^, ^64^, ^65^, ^66^, ^67^, ^68^, ^69^, ^70^, ^71^, ^72^ ^]^ In this regard, further experimental research is required to unravel such complex regulatory networks comprehensively.

In summary, we have built a general framework for the quantification of landscape‐flux and analysis of the underlying mechanism of the cell cycle via nonequilibrium dynamics and thermodynamics based on single‐cell transcriptomic data that can be applied to numerous biological systems. However, it's essential to acknowledge the inherent limitations of our approach and the potential avenues for future research: The mathematical model behind RNA velocity quantity describes only the biological transformation from DNA to RNA, which does not fully capture the intricacies of post‐translational regulation from RNA to protein and protein phosphorylation. Corresponding to landscape‐flux, the flux driving force may become smaller when protein phosphorylation is lacking. It is worthwhile noticing although some details are missing, the main topology of the Mexican landscape and corresponding flux is similar to the protein level studies.^[^ ^9^ ^]^ This implies that the global nature of the cell cycle network may have already been captured at the transcription level. Of course, more information will need to be complemented at the protein level to study the detailed physical mechanisms. While we inferred gene regulatory networks based on differentially expressed genes, this approach might not capture all the actual biological modulation relationships in their entirety. Gene expression and regulation can be influenced by various factors, including chromatin structure, epigenetic modifications, feedback loops, and external stimuli. This prompts a multi‐omics approach for improvement in the future. For further research in the future, we need to combine proteomics to evaluate protein velocity and even combine epigenomics with chromatin velocity to understand the regulation mechanism of the cell cycle more comprehensively. More broadly, when coupled with remarkable advances in single‐cell approaches, including multi‐velocity,^[^ ^89^ ^]^ construction of vector fields,^[^ ^36^ ^]^ as well as spatial multi‐omics,^[^ ^90^ ^]^ we will enable a deeper, quantitative understanding of the spatiotemporally underlying mechanism of the cell cycle and other biological systems via nonequilibrium dynamics and thermodynamics to help prevent various diseases, including cancer and neurodegeneration, and open up new possibilities for precision medicine.

## 4 Experimental Section

### Single‐Cell Sequencing Data Processing and Analysis

1) The scRNA‐seq data for the U2OS‐FUCCI cells (*n* = 1152 cells) were collected for each cell using the SMART‐seq2 extraction and library preparation protocol on an Illumina HiSeq 2500 sequencer, we followed the snake make pipeline at https://github.com/CellProfiling/FucciSingleCellSeqPipeline to perform scRNA‐Seq data preparation and general analysis including filter, dimensionality reduction, clustering analysis and RNA velocity analysis.^[^ ^18^ ^]^ The results used in this analysis were output as TPM values, which were combined into a single data frame containing all protein‐coding genes or isoforms for all cells using a custom script, and then prepared for downstream analysis using Scanpy (version 1.4).^[^ ^91^ ^]^ Cells with fewer than 500 genes detected were filtered (14 cells, 1%) from the analysis, leaving 1138 cells; genes detected in fewer than 100 cells were filtered (6547 genes, 33%), leaving 13 450 protein‐coding genes; and TPM values were log‐transformed. We confirmed that there were no batch effects in these data by using principal component analysis to show no discernible separation of the batches (3 plates with 384 cells each) in the first two principal components. These data were projected onto a UMAP (version 0.3.10),^[^ ^21^ ^]^ which displayed a cyclical arrangement of the cells with different clusters when analyzed by Louvain analysis. 2) For the scEU‐seq data of the RPE1‐FUCCI cells (*n* = 5422 cells), the UMI for a given gene in a cell was considered to be unspliced if at least one base of any read belonging to that UMI mapped outside of the exons of the gene. The minimum number of UMI detected in a cell for the RPE1‐FUCCI cell‐cycle analysis was 2300. For the sake of simplicity, we just focus on the kinetic experimental dataset analysis (*n* = 2793 cells). We used “*dyn.pp.recipe_monocle*” by using Dynamo^[^ ^24^ ^]^ to perform the basic preprocess and cell cycle clustering. 3) For the scRNA‐seq data of the human fibroblasts (*n* = 5367 cells), CellRanger outputs have been processed with velocyto (version 0.17.17),^[^ ^22^ ^]^ analyzed using scanpy (version 1.4.4) ^[^ ^91^ ^]^ and scvelo (version 0.2.2),^[^ ^23^ ^]^ while the imputed spliced and unspliced reads from “*scvelo.pp.moments*” have been used for the analysis. To annotate the different cell cycle phases, we analyze the expression of the cell cycle markers as well as the number of RNA counts per cell.

### RNA Velocity

For the conventional Single‐cell RNA sequencing data, which contains information on both spliced (mature mRNA, exon reads) and unspliced (pre‐mRNA, intron reads) transcripts. Samtools was first used to quantify spliced counts and unspliced counts for each gene. Then, RNA velocity was estimated using Velocyto^[^ ^22^ ^]^ and Scvelo^[^ ^23^ ^]^ toolkits following the protocol under the default parameters. However, the conventional RNA velocity relies on the mispriming of intron reads for current single‐cell platforms, and thus the intron measures were biased and inaccurate, and it was scaled by the splicing rate and lacked real physical meanings (i.e., molecules/hour). The metabolic labeling‐based method which measures both the historical or old and the new and nascent RNA of cells in a controllable way will be a better measurement for RNA velocity and transcriptomic dynamics. *Dynast* toolkit was used for analyzing labeling datasets and quantifying new RNA counts and old RNA counts. Then Dynamo package was used^[^ ^24^ ^]^ to estimate the absolute RNA velocity following the protocol under the default parameters.

### Reconstructing Vector Field from RNA Velocity

Although the estimated RNA velocity could be used to measure the cellular transcriptional dynamics and predict cellular pseudo‐time trajectories, it was a discrete quantity that could not provide a more complete and in‐depth analysis of cellular dynamics and thermodynamics. In principle, a velocity vector field helps to generate hypotheses about how genes regulate cell states and provides a more complete description of how genes regulate each other. Dynamo was used to reconstruct the vector field based on RNA velocity with “*dyn.vf.VectorField*”.^[^ ^24^ ^]^ Then, The divergence, curl, acceleration, and curvature were calculated to perform the differential geometry analysis based on the vector field. It also calculated the jacobian of the vector field to perform genetic perturbation and inference gene regulatory interaction. An analytical function of a vector field was learned from sparse single‐cell samples on the entire space robustly by “*vector_field_function*”, which helps to quantify the nonequilibrium dynamics and thermodynamics of the cell cycle.

### Quantification of Potential Landscape

The stochastic dynamics of the cell cycle system is described by the Langevin equation:

(1) $$\frac{d\mathrm{UMA}P_{i}\left(t\right)}{dt}=\;F\left(\mathrm{UMA}P_{i}\right)+\zeta$$

where UMAP_i_ represents each dimension of UMAP space, which can be projected back to the full transcriptomic space. **F** is the reconstructed vector field function. The noise term ζ adopts the independent additive white Gaussian noise, 〈ζ(*t*)〉 = 0 and 〈ζ(*t*)ζ(*t*′)〉 =  2*D*δ(*t* − *t*′). *D* is the diffusion coefficient matrix. Typically, the noise term is associated with the intensity of cellular fluctuations either from environmental external fluctuations or intrinsic fluctuations. Even in bacterial cells with less number of molecules than in mammalian cells, a study has shown that external fluctuation dominates.^[^ ^34^ ^]^ In mammalian cells, the complex regulations of ≈10000 genes make their stochastic expression dynamics more robust to internal noise. Therefore, the study assumes the intrinsic cellular fluctuations to be effectively averaged out and mainly considers the effects of external fluctuations.

To visualize the global dynamics of the cell cycle, the potential landscape that represents the weights of different possible states or phases was mapped out, starting from enough random initial conditions (initial gene expression states), the system would eventually evolve into different cell cycle phase steady state, which may be monostable, multi‐stable or limit cycle steady state. All steady‐state trajectories in the UMAP space were divided into several small regions, and the steady‐state trajectory density in each small region was counted as steady‐state probability *P_ss_*, which tells how likely a cell was to be in a particular state. It includes the effects of diffusion through the resulting steady‐state probability distribution. *U* is the generalized potential related to the steady‐state probability by *U*  =   − ln *P_ss_*. In simpler terms, this landscape helps us understand the “hills” and “valleys” of cell cycle dynamics, where “hills” represent less likely states and “valleys” represent more likely states.

### Driving Force Decomposition to the Gradient of the Potential Landscape and Curl Flux

The cellular dynamics can be described using the probabilistic evolution of the diffusion equation, ∂*P*/∂*t* + ∇ · **J** (**x**, *t*) =  0, this equation tells how the probability distribution of a system changes over time. The key idea here is that any local change in this distribution is due to a net movement or “flux” of probability in or out of that region. This quantifies how far the system is away from equilibrium with the equilibrium system having zero net input or output. It defines a vector, **J**, that represents the flow of the probability in the cell cycle system. This flow vector is influenced by two main ingredients: the driving force **F** and the gradient of the probability distribution. So the probability flux vector **J** of the system in concentration space **x** is defined as $J\;\left(x,t\right)=\;FP-D\cdot \frac{\partial }{\partial x}P$.

In general, the dynamic driving force **F** can be decomposed into a gradient of the potential and a curl flow flux:^[^ ^7^ ^]^

(2) $$F\;=\frac{D}{P_{ss}}\cdot \frac{\partial }{\partial x}P_{ss}+\;\frac{J_{\mathrm{ss}}\left(x\right)}{P_{ss}}=\;-D\frac{\partial }{\partial x}U+\frac{J_{\mathrm{ss}}\left(x\right)}{P_{ss}}$$

*P_ss_* represents steady‐state probability distribution and potential landscape U is defined as U  =   − ln *P_ss_*. In systems at equilibrium steady state, there's no net movement, $\frac{\partial P_{ss}}{\partial t}=\;0$, thus ∇ ·  **J**_**ss**_ =  0. With detailed balance, there is no net flux and **J**_**ss**_ =  0, the gradient of potential controls the underlying dynamics as the driving force. For nonequilibrium systems, there's a net flow even at steady state, although ∇ ·  **J**_**ss**_ =  0, the net flux **J**_**ss**_ ≠ 0. Therefore, both the gradient of potential and nonzero steady‐state probability net flux of probability together determine the dynamics and global properties. In the study, the cell cycle was a nonequilibrium system. The dynamics of a nonequilibrium network can be described as a spiral, along the gradient direction, not as the case of the equilibrium state only following the gradient. It was similar to the electrons moving in both electric and magnetic fields.^[^ ^7^ ^]^

### EPR (Entropy Production Rate)

In a nonequilibrium open system, there were constant exchanges in energy and information with their surroundings, which resulted in the lost or dissipation of energy. The dissipation gives a global physical characterization of the nonequilibrium system. In the steady state, the dissipation of energy was closely associated with the entropy production rate (EPR). Entropy was a measure of the disorder or randomness in a system and the entropy formula for the system was well‐known,^[^ ^92^ ^]^

(3) $$S=-k_{B}\int P\left(x,t\right)\ln P\left(x,t\right)dx$$

where *k*_B_ is the Boltzmann constant, and *P*(**x**, *t*) is the probability distribution of the system.

By differentiating the above function, it can determine how entropy changes over time, the increase of the entropy at constant temperature *T* is shown as follows:

(4) $$\begin{array}{ccc} T\;\dot{S} & & =k_{B}\;*T\int \left(\ln P+1\right)\nabla \cdot J\;dx\\ & & =\;-\int \left(k_{B}T\nabla \ln P-F\right)\cdot J\;dx-\int F\cdot J\;dx \end{array}$$

where $-\int (k_{B}T\nabla \ln P-F)\cdot J\;dx\;=\;EPR$ is the entropy production rate, and ∫F·Jdx=HDR is the mean rate of heat dissipation. In steady state, $\dot{S}=0$0, and the entropy production rate (EPR) was equal to the heat dissipation rate (HDR). In this article, the heat dissipation rate was calculated at a steady state and also verified that it was the same numerically as the entropy production rate at a steady state.

### Flux Integration

The curl flux obtained by decomposing the driving force was a discrete vector. In order to better quantify the curl probabilistic flux of the cell cycle oscillation, *Flux_Loop_* was defined as the flux integration along the looping trajectory ( $Flux_{Loop}=\oint_{0}^{L}J\cdot dl/\oint_{0}^{L}dl\;$) of the cell cycle oscillation divided by the loop length in gene expression space. From **J** = **F** *P* − **D** · ∇*P*, it have $\oint_{0}^{L}J\cdot dl=\oint_{0}^{L}FP\cdot dl\;-D\cdot \;\oint_{0}^{L}\nabla P\cdot dl=\oint_{0}^{L}FP\cdot dl\;$. The flux integration was calculated in 2D UMAP space projection.

### In Silico Perturbation

When genes in cells were altered or “perturbed,” it could influence the cells' behavior, specifically their velocity or vector field. For small perturbations (Δ**x**) of the gene expression in cells, specific to the influence of perturbation dx_1_, dx_2_, …, dx_n_ of each gene on velocity or vector field, it can be calculated with the exact differential:

(5) $${\mathrm{df}}_{i}=\frac{\partial f_{i}}{\partial x_{1}}{\mathrm{dx}}_{1}+\frac{\partial f_{i}}{\partial x_{2}}{\mathrm{dx}}_{2}+\cdot \cdot \cdot +\frac{\partial f_{i}}{\partial x_{n}}{\mathrm{dx}}_{n}$$

which calculates the change in velocity based on the change in the gene expressions. In simpler terms, it tells how much a small change in a gene will influence the cell's state dynamics.

In vectorized form:

(6) Δf=JΔx

**J** is the Jacobian matrix of the vector field and Δ**f** is the velocity and vector field response in silico genetic perturbation. Sometimes, the change in the cell velocity might be too small to be noticed. To make it more evident, one can amplify this change using a proportionality constant, which helps to see the effects more clearly. In practice, a proportionality constant *c* is often added to the perturbation Δ**x** to amplify the response Δ**f**.

The “*dyn.vf.perturbation*” function was first used to perform in silico perturbation and visualize the RNA velocity from the perturbation effect vectors. Then, reconstruct the perturbed vector field from the perturbed RNA velocity with “*dyn.vf.vectorfield*” function. Finally, it performs a random dynamics simulation on the vector field of the genetic perturbation to obtain the perturbed landscape, and then quantify the relevant flux and EPR.

### LAPs (Least Action Paths)

Following the approaches based on the Freidlin–Wentzell theory,^[^ ^93^ ^]^ the most probable transition path from attractor j at time 0 to attractor *k* at time *T*, could be acquired by minimizing the action functional over all possible paths:

(7) $$S_{T}\left(x_{\mathrm{jk}}\right)=\frac{1}{2}\int_{0}^{T}\frac{{\left|{\dot{x}}_{\mathrm{jk}}-F\left(x_{\mathrm{jk}}\right)\right|}^{2}}{D}dt$$

where *D* is the diffusion coefficient accounting for the stochasticity of gene expression, and for simplicity here it was assumed to be a constant. This path **x** is called the least action path (LAP). Specifically, the optimal path between any two cell states was searched by variating the continuous path connecting the source state to the target while minimizing its action and updating the associated transition time. In the single‐cell data analysis, the LAPs are pseudo‐temporal paths.

### MFPT (Mean First Passage Time)

For rare transitions with *S_T_* ≫ 0, the transition rate (number of transitions per unit time) is proportional to the exponential of the actions. The Freidlin–Wentzell theorem dictates that the LAP with the minimal traversal time (which will be referred to as the optimal path below) contributes the most to this transition rate:^[^ ^93^ ^]^

(8) $$R\left(A\to B\right)\approx C\;\exp\left(-S_{T}\right)$$

where *A* and *B* are two cell cycle phases, *S*_T_ is the action of the optimal path, and *C* a proportional factor. Furthermore, the transition time, or more specifically MFPT, is related to the transition rate:

(9) $$\mathrm{MFPT}\;=\frac{1}{R\left(A\to B\right)}\;$$

### Phase Diffusion and Coherence Time

The 500 trajectories starting with the same initial condition were simulated. For the *j*‐th trajectory, we obtained its *i*‐th peak time *t_ij_* from the trajectory *x_j_* (*t*) after smoothing (smooth function in MATLAB was used). The peak positions for two trajectories are shown in Figure S7 (Supporting Information). For each of the trajectories, it computed the mean of their *i*‐th peak time $m_{i}\;=\sum_{j}t_{\mathrm{ij}}/N\;$, and variance $\sigma_{i}^{2}\;=\sum_{j}{\left(t_{\mathrm{ij}}-m_{i}\right)}^{2}/\left(N-1\right)\;$, where *N* is the total number of trajectories. The average period *T* is given by *T*  = *m*_i_ /*i*. Asymptotically, $\sigma_{i}^{2}$ depends linearly on *m*_i_, and the slope of this linear dependence is the peak‐time diffusion constant *D*, which has the dimension of time. Over time, the cell cycle may not always oscillate with the same timing. Phase diffusion measures how much the timing of these oscillations spreads out over time. A higher phase diffusion means that the cells were less consistent in their timing. The phase diffusion constant *D*_∅_ is linearly proportional to *D*:^[^ ^50^ ^]^

(10) $$D_{\emptyset }=\;D\frac{{\left(2\pi \right)}^{2}}{T}$$

The autocorrelation function was used to measure how similar a cell's behavior is at two different times:^[^ ^50^ ^]^

(11) $$\begin{array}{ccc} C\;\left(t\right) & & =\frac{\langle \left(x\left(t+s\right)-\langle x\rangle \right)\left(x\left(s\right)-\langle x\rangle \right)\rangle }{\langle x^{2}\rangle -{\langle x\rangle }^{2}}\\ & & =\exp\left(-\frac{t}{\tau_{c}}\right)\;\times \cos\left(\frac{2\pi t}{T}\right) \end{array}$$

where *s* is a time variable, the period is given by *T* and τ_*c*_ is the coherence time, a measure of how consistent the oscillations were over time. If the cells oscillate very consistently, they have a long coherence time. If their oscillations were more random, the coherence time was short.

### Power Spectral Density (PSD) from Autocorrelation

The distribution of average power of a signal *x*(*t*) in the frequency domain ω is called the power spectral density (PSD) or power density (PD) or power density spectrum, which helps to understand the strength and the distribution of frequencies in the cell cycle oscillation signal. To derive the PSD function, consider a power signal as a limiting case of an energy signal, i.e., the signal *Z*(*t*) is zero outside the interval |τ/2|, the signal *Z*(*t*) is given by,

(12) $$Z\;\left(t\right)=\left\{\begin{array}{c} x\left(t\right),\;\left|t\right|<\frac{\tau }{2}\\ 0,\;otherwise \end{array}\right.\;$$

where *x*(*t*) is a power signal of the same magnitude extending to infinity.

As the signal *Z*(*t*) is a finite duration signal of duration *τ* and thus, it is an energy signal having energy E, which is given by,

(13) $$E=\int_{-\infty }^{\infty }{\left|Z\left(t\right)\right|}^{2}dt\;=\frac{1}{2\pi }\;\int_{-\infty }^{\infty }{\left|Z\left(\omega \right)\right|}^{2}d\omega$$

where $Z\left(t\right)\overset{FT}{↔}Z\left(\omega \right)$. Also,

(14) $$\int_{-\infty }^{\infty }{\left|Z\left(t\right)\right|}^{2}dt=\int_{-\frac{\tau }{2}}^{\frac{\tau }{2}}{\left|x\left(t\right)\right|}^{2}dt\;$$

Therefore, we have,

(15) $$\frac{1}{\tau }\int_{-\frac{\tau }{2}}^{\frac{\tau }{2}}{\left|x\left(t\right)\right|}^{2}dt=\frac{1}{2\pi }\;\cdot \frac{1}{\tau }\int_{-\infty }^{\infty }{\left|Z\left(\omega \right)\right|}^{2}d\omega$$

Hence, when τ → ∞, then the LHS of the above equation gives the average power (P) of the signal *x*(*t*), i.e.,

(16) $$P\;=\frac{1}{2\pi }\int_{-\infty }^{\infty }\lim_{\tau \to \infty }\left(\frac{{\left|Z\left(\omega \right)\right|}^{2}}{\tau }\right)d\omega$$

if τ → ∞, then $\left(\frac{{\left|Z(\omega )\right|}^{2}}{\tau }\right)$ approaches a finite value. Assume this finite value is represented by *S*(ω), i.e.,

(17) $$S\;\left(\omega \right)=\lim_{\tau \to \infty }\frac{{\left|Z\left(\omega \right)\right|}^{2}}{\tau }\;$$

this expression is called the power spectral density (PSD) of the signal *Z*(*t*). Therefore, for the function *x*(*t*), the PSD function is given by,

(18) $$S\;\left(\omega \right)=\lim_{\tau \to \infty }\frac{{\left|x\left(\omega \right)\right|}^{2}}{\tau }\;$$

The power spectral density function *S*(ω) and the autocorrelation function *R*(τ) of a power signal form a Fourier transform pair, i.e.,

(19) $$R\left(\tau \right)\overset{FT}{↔}S\left(\omega \right)$$

### Phase Coherence

The robustness of the oscillation with respect to the diffusion coefficient *D* can be quantified further by the phase coherence ξ, which measures the degree of periodicity of the time evolution of a given variable.^[^ ^52^ ^]^ The phase coherence ξ quantitatively measures the degree of persistence of the oscillatory phase and is defined as follows. First, the vector *N* (*t*) = *n*_1_ (*t*)*e*_1_ + *n*_2_(*t*)*e*_2_ is shown in Figure S12I (Supporting Information). The unit vectors are *e*_1_ = (1,0) and *e*_2_ = (0, 1), and *n*_1_(*t*) and *n*_2_(*t*) are the concentration of the two kinds of protein molecules at time *t*. Then, ϕ(*t*) is the phase angle between *N*(*t*) and *N*(*t* + τ), where τ should be smaller than the deterministic period and larger than the fast fluctuations. ϕ(*t*) > 0 to represent that the oscillation goes on the positive orientation (counterclockwise). The formula of ξ is shown as

(20) $$\xi \;=\frac{2\sum_{i}\theta \left(\phi \left(t\right)\right)\phi \left(t\right)}{\sum_{i}\left|\phi \left(t\right)\right|}\;-1$$

where θ(ϕ) = 1 when ϕ(*t*) > 0, and θ(ϕ) = 0 when ϕ(*t*) ≤ 0, and sums were taken over every time step for the simulant trajectory. ξ ≈ 0 means the system moves stochastically and has no coherence. The oscillation is most coherent as ξ is close to 1.

### Gene Regulatory Network Inference

To construct the gene interaction network of the cell cycle, it first calculated and obtained all differentially expressed genes in different cell cycle phases (Tables S1 and S2, Supporting Information). After that, the interactions between the differentially expressed genes were calculated by Jacobian based on the reconstructed vector field function. Positive values from Jacobian represent activation and negative values represent inhibition.^[^ ^24^ ^]^ The visualization of all networks was performed using Cytoscape.^[^ ^94^ ^]^ The nodes and edges each represent the genes and interactions in a network.

To infer cell cycle phase‐specific gene regulatory networks (GRNs) following the cell differentiation, it utilizes spliceJAC^[^ ^57^ ^]^, which uses RNA velocity to estimate the Jacobian of the gene–gene interaction matrices, inferring the state‐specific regulatory interactions. The top 20 genes of each cell cycle phase were selected, which were the variables in the regression problem, to be smaller than the number of cells, which were the observations.

### Estimating Kinetic Parameters by Fitting the Jacobian versus Expression Curve

The Hill function was a mathematical model commonly used to describe various regulatory interactions in genetic and biochemical networks. Because the reconstructed vector field was expressed as a set of implicit basis functions, not explicitly as Hill functions, in the current framework it was not able to directly obtain kinetic parameters such as the Hill coefficient. Nevertheless, the reconstructed vector field encodes such information, and additional computations were applied to extract that information.^[^ ^24^ ^]^ This possibility on simplistic network motifs such as CCNE2‐CENP2 and CCNE2‐KIF23 was demonstrated, by fitting the derivatives of inhibitory or activation Hill equations to the corresponding Jacobian elements. Further efforts would be needed to make such efforts generally applicable to systems with more sophisticated mechanisms.

Formally, it assumes that the activation effect of gene x on the target gene y takes the form of an activating Hill function:

(21) $$x\to y:\;\frac{Ax^{n}}{\left(K+x^{n}\right)}$$

and that the inhibition effect assumes the form of an inhibitory Hill function:

(22) $$x⊣y:\;\frac{AK}{\left(K+x^{n}\right)}$$

For the activation‐inhibition and self‐activation of two genes x and y, the ODEs can be written as:

(23) $$\begin{array}{c} \frac{dx}{dt}=f_{x}=\frac{A_{1}x^{n1}}{\left(K_{1}+x^{n1}\right)}+\frac{A_{2}K_{2}}{\left(K_{2}+y^{n2}\right)}-g_{1}\times x\\ \frac{dy}{dt}=f_{y}=\frac{A_{3}x^{n3}}{\left(K_{3}+y^{n3}\right)}+\frac{A_{4}x^{n4}}{\left(K_{4}+x^{n4}\right)}-g_{2\;}\times y \end{array}$$

The Jacobian of this system is a 2‐by‐2 matrix:

(24) $$J\;=\left[\begin{array}{cc} \frac{\partial f_{x}}{\partial x} & \frac{\partial f_{x}}{\partial y}\\ \frac{\partial f_{y}}{\partial x} & \frac{\partial f_{y}}{\partial y} \end{array}\right]\;=\left[\begin{array}{cc} \frac{A_{1}K_{1}n_{1}x^{n_{1}-1}}{{\left(K_{1}+x^{n_{1}}\right)}^{2}}-g_{1} & -\frac{A_{2}K_{2}n_{2}y^{n_{2}-1}}{{\left(K_{2}+y^{n_{2}}\right)}^{2}}\\ -\frac{A_{3}K_{3}n_{3}x^{n_{3}-1}}{{\left(K_{3}+x^{n_{3}}\right)}^{2}} & \frac{A_{4}K_{4}n_{4}x^{n_{4}-1}}{{\left(K_{4}+y^{n_{4}}\right)}^{2}}-g_{2} \end{array}\right]$$

In Figure 6E and Figure S14E (Supporting Information), the means and standard deviations of the Jacobian versus expression profiles were calculated and fitted with the above derivatives using the Dynamo: “*dyn.pl.response*”, which could fit and obtain the parameters of hill function (Table S3, Supporting Information).

### Quantification and Statistical Analysis

The calculation of the amplitude and period of cell cycle oscillations was averaged multiple times using MATLAB. All correlation analyses and confidence intervals were calculated in Origin. The fitting evaluation of phase diffusion was performed by MATLAB.

## Conflict of Interest

The authors declare no conflict of interest.

## Author Contributions

L.Z. and J.W. performed conceptualization, methodology, investigation, visualization, and wrote, reviewed, and edited the final manuscript. J.W. performed supervision. All authors read and approved the final manuscript.

## Supporting information

Supporting Information

## Data Availability

The scRNA‐seq raw data of U2OS‐FUCCI cell cycle from the paper by Mahdessian et al,^[^ ^18^ ^]^ which are available at GEO with accession GSE146773. The scEU‐seq data of RPE1‐FUCCI cell cycle from the paper by Battich et al,^[^ ^19^ ^]^ which are available at GEO with accession number GSE128365. The scRNA‐seq raw data of the human fibroblast cell cycle from the paper by Riba et al,^[^ ^20^ ^]^ which are available at GEO with accession GSE167609. The processed data and source codes used in this study are publicly available from Github (https://github.com/Zhu‐1998/cellcycle).
