# Supplementary material for: Quantifying Landscape‐Flux via Single‐Cell Transcriptomics Uncovers the Underlying Mechanism of Cell Cycle
Source: Adv Sci (Weinh). 2024 Feb 14;11(16):2308879. doi: 10.1002/advs.202308879 (PMC11040366; doi:10.1002/advs.202308879)
Supplement: Supplementary file 1 — Supporting Information [file ADVS-11-2308879-s001.pdf]

ADVANCED SCIENCE
Open Access

Supporting Information

for *Adv. Sci.*, DOI 10.1002/advs.202308879

Quantifying Landscape-Flux via Single-Cell Transcriptomics Uncovers the Underlying Mechanism of Cell Cycle

*Ligang Zhu and Jin Wang**

Supporting Information

Quantifying Landscape-Flux via Single-Cell Transcriptomics Uncovers the Underlying Mechanism of Cell Cycle

*Ligang Zhu, and Jin Wang**

Supplementary Tables

Table S1. Differential expression genes for U2OS cell cycle regulation.

test	gene	mean_exp_t	vers	mean_exp_v	log2_fc	pval	qval
**G1-S**	*CCNE2*	21.688	S	9.627	1.172	2.64E-16	1.33E-15
	MSH6	75.596	G2-M	27.095	1.480	1.13E-63	9.91E-62
	MCM10	40.248	G2-M	15.695	1.359	6.55E-37	5.05E-36
	MCM3	149.996	G2-M	43.955	1.771	1.85E-71	4.84E-69
	CDC6	140.648	G2-M	43.638	1.688	4.11E-61	1.54E-59
	SLBP	70.472	G2-M	21.630	1.704	5.23E-60	1.25E-58
	UNG	60.413	G2-M	13.771	2.133	2.23E-62	9.73E-61
	HELLS	15.808	G2-M	6.339	1.318	1.32E-24	8.06E-24
	RAD51	42.229	G2-M	13.430	1.653	9.65E-34	7.23E-33
**S**	*MCM6*	24.616	G2-M	9.118	1.433	5.60E-30	5.31E-29
	SYBU	2.759	G2-M	0.939	1.555	8.43E-03	1.11E-02
	RASGEF1B	0.859	G2-M	0.355	1.275	4.32E-02	4.97E-02
**G2-M**	*PSRC1*	128.138	G1-S	22.815	2.490	1.27E-51	2.04E-50
	CCNF	70.053	G1-S	14.102	2.313	2.62E-47	3.31E-46
	KIF18A	33.132	G1-S	9.076	1.868	4.85E-35	3.80E-34
	FAM83D	198.470	G1-S	23.666	3.068	6.35E-49	8.77E-48
	CENPE	48.301	G1-S	11.506	2.070	4.32E-33	2.91E-32
	NEK2	75.842	G1-S	16.379	2.211	1.63E-35	1.31E-34
	CENPA	48.093	G1-S	12.087	1.992	5.63E-33	3.71E-32
	GAS2L3	31.991	G1-S	7.663	2.062	3.53E-24	1.50E-23
	BORA	45.495	G1-S	7.906	2.525	4.58E-34	3.24E-33
	CDC25C	29.066	G1-S	7.742	1.909	7.03E-22	2.68E-21
	KIF20A	107.657	S	32.612	1.723	8.60E-33	5.42E-32
	FAM72D	8.484	S	3.734	1.184	1.03E-06	2.57E-06
**M**	*PIF1*	22.567	G1-S	1.796	3.651	5.90E-10	6.23E-10
	H2BC6	1.740	G2-M	0.442	1.977	2.59E-02	2.68E-02
**M-G1**	*PDE4B*	2.824	G2-M	0.736	1.940	7.50E-03	7.77E-03

Table S2. Differential expression genes for RPE1 cell cycle regulation.

test	gene	mean_exp_t	vers	mean_exp_v	log2_fc	pval	qval
**G1-S**	*CCNE2*	0.949	M-G1	0.337	1.494	6.65e-25	1.77e-24
**S**	*H4C3*	13.074	M-G1	4.086	1.678	1.87e-67	4.49e-66
	AURKB	3.018	M-G1	1.262	1.258	8.96e-39	7.16e-38
	CDK1	1.926	M-G1	0.709	1.442	8.75e-36	5.25e-35
**G2-M**	*TOP2A*	9.015	G1-S	2.390	1.915	4.68e-52	1.76e-50
	KIF23	2.5	G1-S	0.855	1.548	1.51e-30	1.22e-29
	HJURP	2.241	G1-S	0.602	1.896	8.81e-21	3.43e-20
	KPNA2	3.265	G1-S	0.895	1.867	2.22e-31	2.09e-30
**M**	*VEGFA*	0.853	G1-S	0.385	1.148	1.79e-06	2.81e-06
	TPX2	3.857	G1-S	1.438	1.423	1.80e-48	3.23e-47
	CKAP2	1.958	G1-S	0.720	1.443	1.20e-23	3.61e-23
	GAS2L3	1.291	G1-S	0.503	1.360	1.18e-12	2.23e-12
	CDC20	3.386	G1-S	0.940	1.849	1.96e-52	4.71e-51
**M-G1**	*MATR3*	0.299	G2-M	0.141	1.084	1.28e-07	2.99e-07
	KIF20A	1.137	G1-S	0.502	1.179	1.43e-23	4.99e-23
	CCNB1	0.734	G1-S	0.327	1.166	4.92e-14	1.29e-13

Table S3. The hill function and parameters of gene interaction.

ODEs	Interaction term	Parameters
The interaction between *CCNE2* and *CENPE*		
$\frac{d[CCNE2]}{dt} = f_{CCNE2} = V_1 + V_2 - V_3$	Self-activation<br>$V_1 = \frac{A_1[CCNE2]^{n1}}{(K_1 + [CCNE2]^{n1})}$	A_1 =3.7e-3<br>K_1 =16.2<br>n_1 =2.7
	CENPE activates CCNE2<br>$V_2 = \frac{A_3[CENPE]^{n3}}{(K_3 + [CENPE]^{n3})}$	A_3 =9.3e-3<br>K_3 =83.9<br>n_3 =4.1
	Degradation<br>$V_3 = g_1 \cdot [CCNE2]$	g_1 =3.4e-15
$\frac{d[CENPE]}{dt} = f_{CENPE} = V_4 + V_5 - V_6$	Self-activation<br>$V_4 = \frac{A_2[CENPE]^{n2}}{(K_2 + [CENPE]^{n2})}$	A_2 =1.3e-2<br>K_2 =37.9<br>n_2 =3.1
	CCNE2 inhibits CENPE<br>$V_5 = \frac{A_4K_4}{(K_4 + [CCNE2]^{n4})}$	A_4 =9.8e-4<br>K_4 =5.1<br>n_4 =2.6
	Degradation<br>$V_6 = g_2 \cdot [CENPE]$	g_2 =2.0e-4
The interaction between *CCNE2* and *KIF23*		
$\frac{d[CCNE2]}{dt} = f_{CCNE2} = V_1 + V_2 - V_3$	Self-activation<br>$V_1 = \frac{A_1[CCNE2]^{n1}}{(K_1 + [CCNE2]^{n1})}$	A_1 = 4.7e-4<br>K_1 =2.3<br>n_1 =1.1
	KIF23 inhibits CCNE2<br>$V_2 = \frac{A_4K_4}{(K_4 + [KIF23]^{n4})}$	A_4 =3.2e-4<br>K_4 =0.9<br>n_4 =3.1
	Degradation<br>$V_3 = g_1 \cdot [CCNE2]$	g_1 =1.3e-4
$\frac{d[KIF23]}{dt} = f_{KIF23} = V_4 + V_5 - V_6$	Self-activation<br>$V_4 = \frac{A_2[KIF23]^{n2}}{(K_2 + [KIF23]^{n2})}$	A_2 =1.5e-4<br>K_2 =1.7<br>n_2 =6.6
	CCNE2 activates KIF23<br>$V_5 = \frac{A_3[CCNE2]^{n3}}{(K_3 + [CCNE2]^{n3})}$	A_3 =4.1e-4<br>K_3 =1.2<br>n_3 =2.4
	Degradation<br>$V_6 = g_2 \cdot [KIF23]$	g_2 =6.7e-27

Supplementary Figures

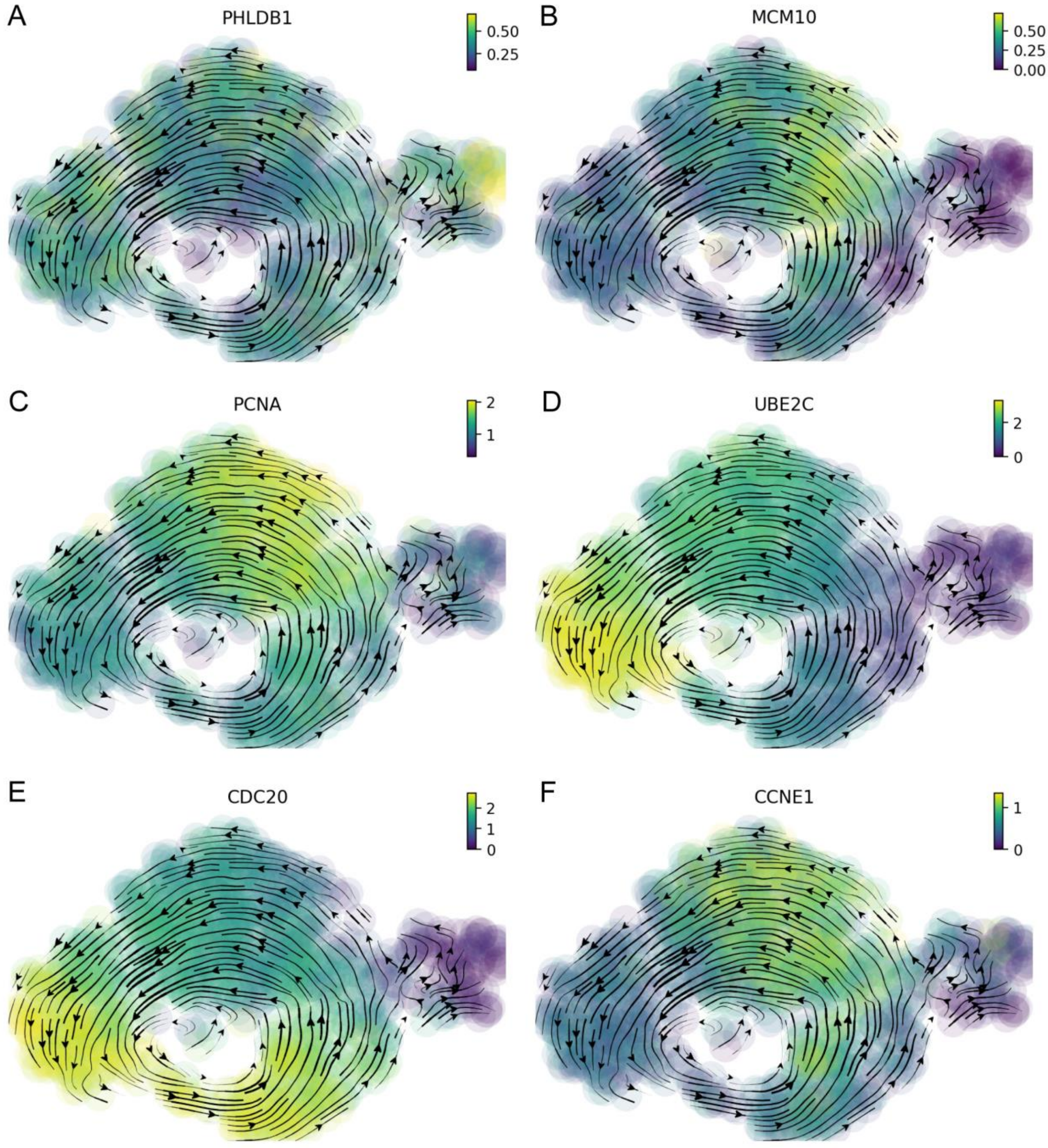

Figure S1. The key gene expression of different cell cycle phases. (A) *PHLDB1* has high expression in the G0 phase. (B) *MCM10* has high expression in the G1-S phase. (C) *PCNA* has high expression in the S phase. (D) *UBE2C* has high expression in the G2-M phase. (E) *CDC20* has high expression in the M phase. (F) *CCNE1* has high expression in the M-G1 phase.

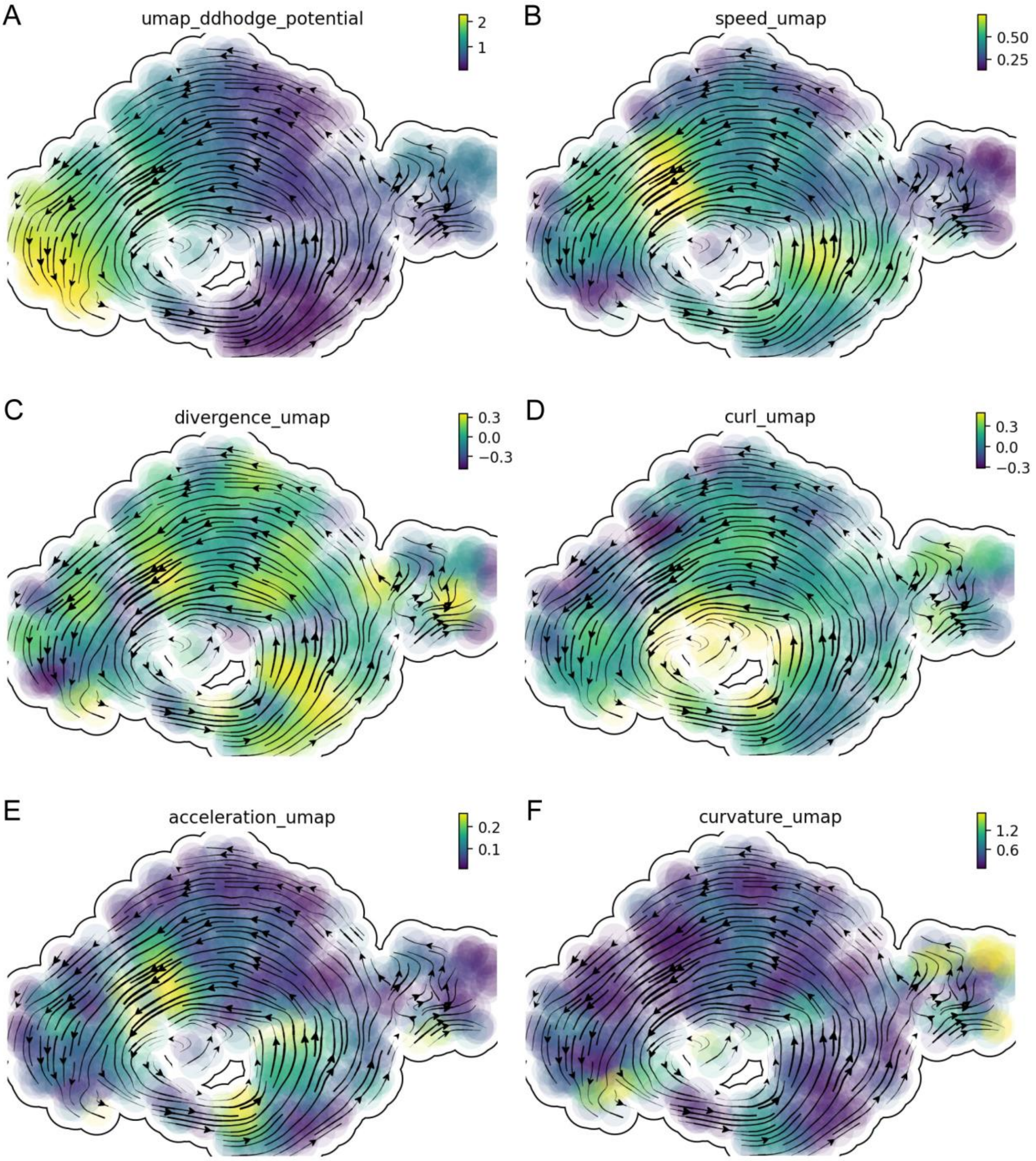

Figure S2. The differential geometric analysis of cell cycle dynamics of U2OS cells. (A) Ddhodge potential of the reconstructed vector field among all cell cycle phases. (B) Same as in (A) but for the speed. (C) Same as in (A) but for the divergence. (D) Same as in (A) but for the curl. (E) Same as in (A) but for the acceleration. (F) Same as in (A) but for the curvature.

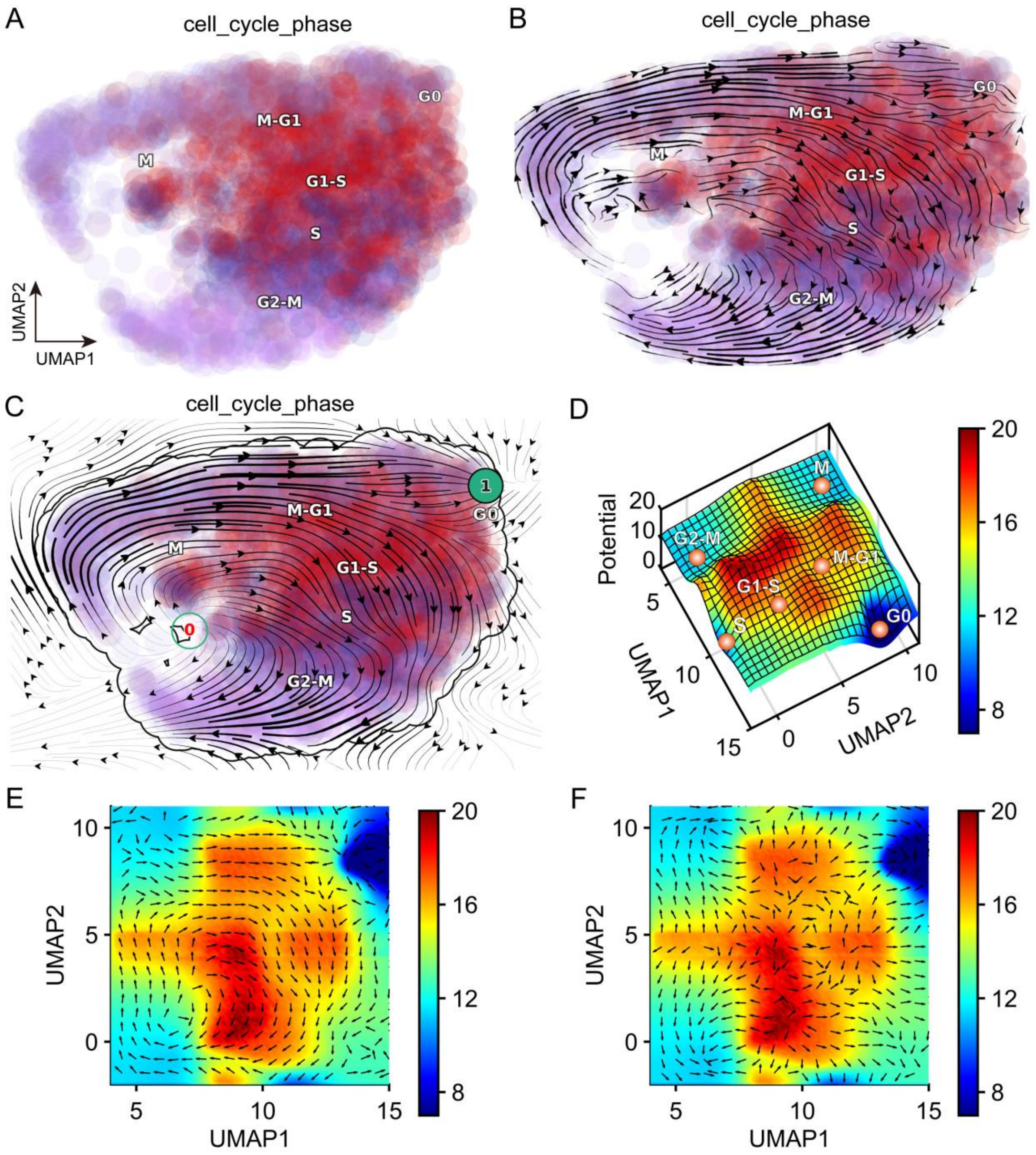

Figure S3. Constructing cell cycle landscape-flux by scEU-seq data of RPE1 cells. (A) Cell cycle phase clusters by UMAP. (B) RNA velocity of cell cycle dynamics. (C) Reconstructed vector field of cell cycle dynamics. The red digit 0 to represent the limit cycle attractor, reflecting cell cycle oscillations, and the black digit 1 to represent the absorption fixed-point attractor, reflecting the stationary phase of the cell at G0. (D) Potential landscape of cell cycle dynamics in UMAP. (E) Curl flux (black arrow) of cell cycle dynamics landscape in UMAP. (F) The gradient force (black arrow) of cell cycle dynamics landscape in UMAP.

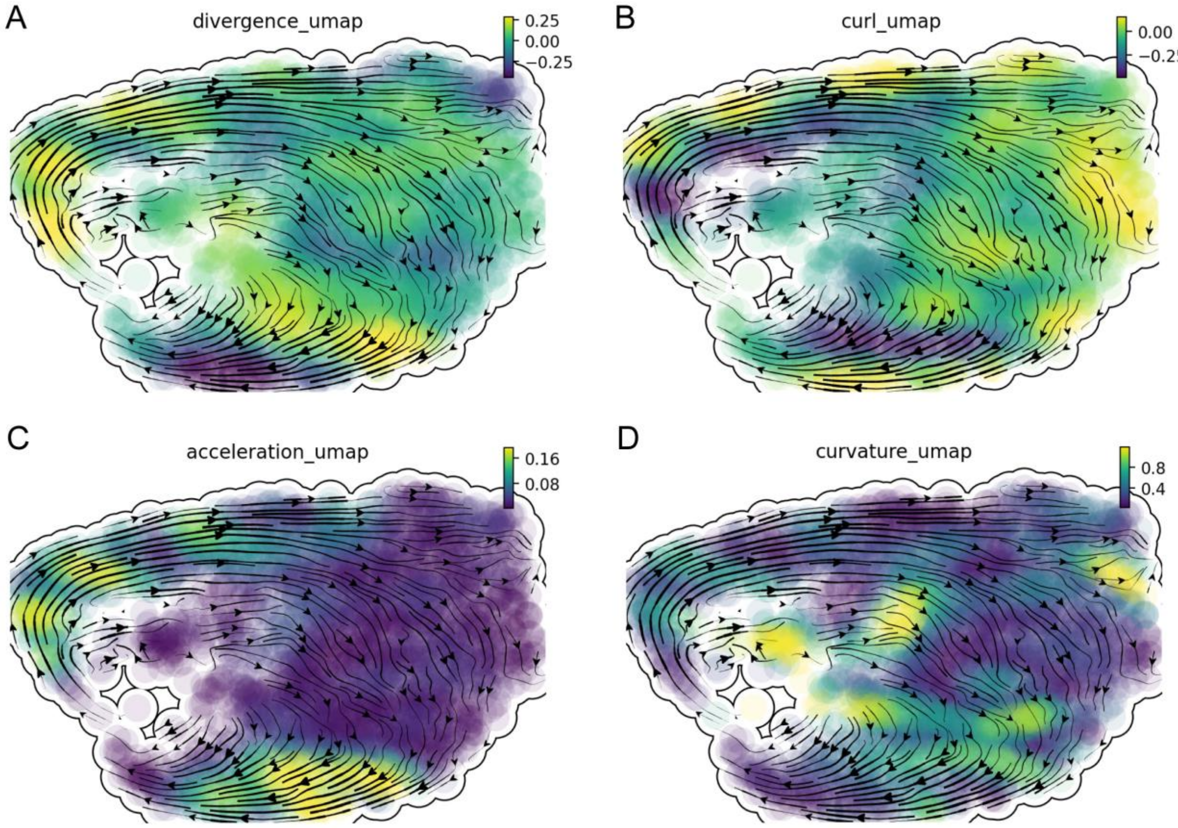

Figure S4. The differential geometric analysis of cell cycle dynamics of RPE1 cell. (A) Divergence of the reconstructed vector field among all cell cycle phases. (B) Same as in (A) but for the curl. (C) Same as in (A) but for the acceleration. (D) Same as in (A) but for the curvature.

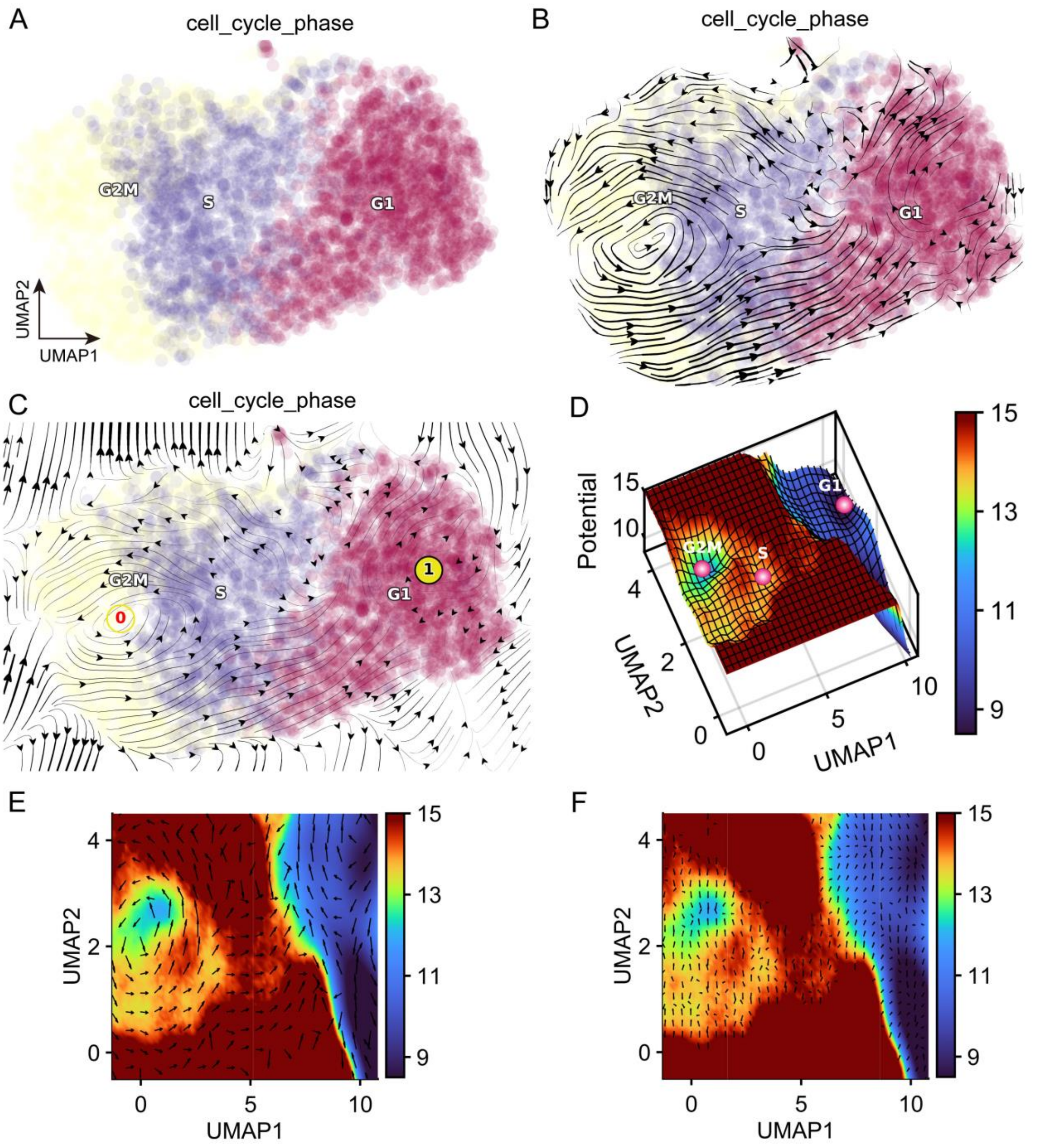

Figure S5. Constructing cell cycle landscape-flux by scRNA-seq data of human fibroblasts. (A) Cell cycle phase clusters by UMAP. (B) RNA velocity of cell cycle dynamics. (C) Reconstructed vector field of cell cycle dynamics. The red digit 0 to represent the limit cycle attractor, reflecting cell cycle oscillations, and the black digit 1 to represent the absorption fixed-point attractor, reflecting the stationary phase of the cell at G1. (D) Potential landscape of cell cycle dynamics in UMAP. (E) Curl flux (black arrow) of cell cycle dynamics landscape in UMAP. (F) The gradient force (black arrow) of cell cycle dynamics landscape in UMAP.

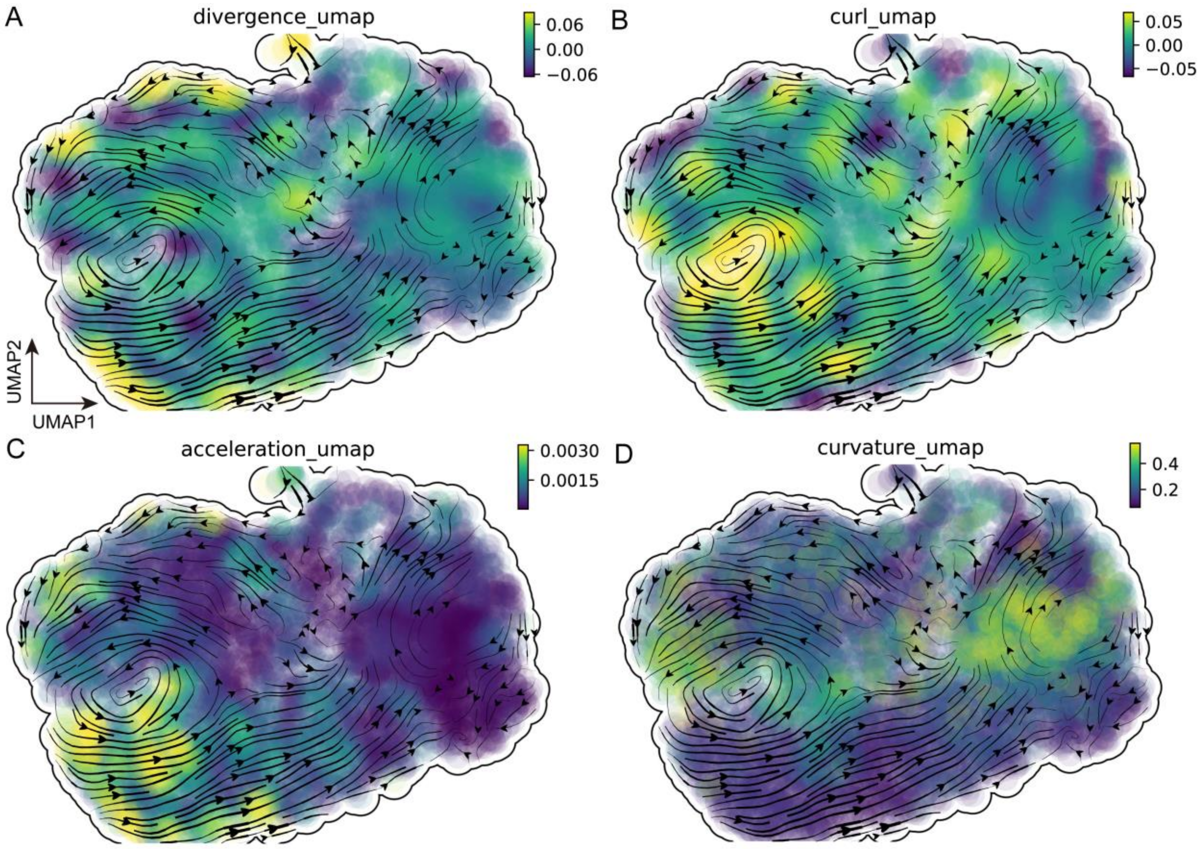

Figure S6. The differential geometric analysis of cell cycle dynamics of human fibroblasts. (A) Divergence of the reconstructed vector field among all cell cycle phases. (B) Same as in (A) but for the curl. (C) Same as in (A) but for the acceleration. (D) Same as in (A) but for the curvature.

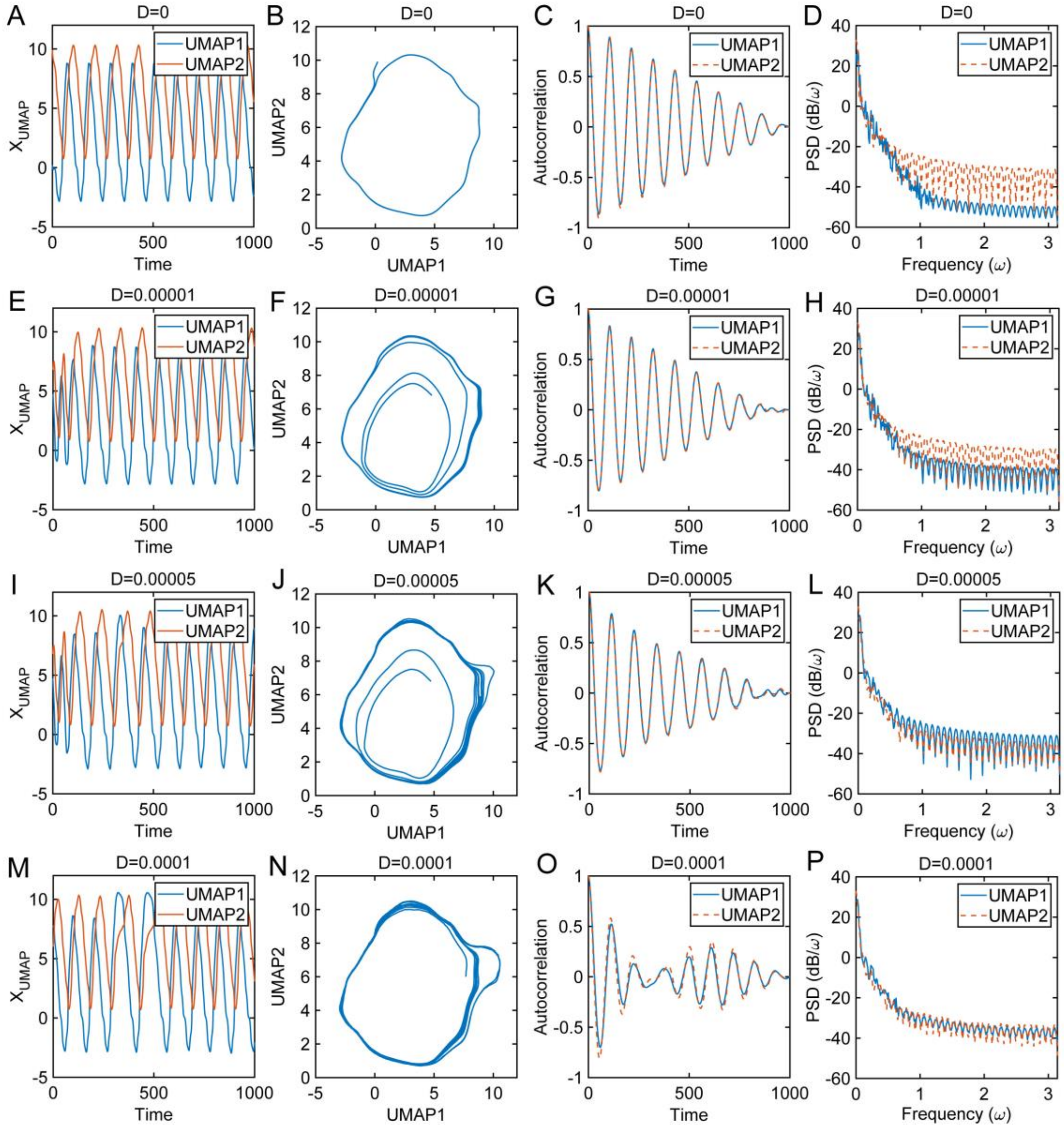

Figure S7. The trajectory, autocorrelation and power spectral density of cell cycle oscillation dynamics with different strength of noise. (A) The trajectories along time of the cell cycle for different diffusion coefficient D=0. (B) The trajectories in phase space corresponding to (A) for different diffusion coefficient D=0. (C) The autocorrelation corresponding to (A) for different diffusion coefficient D=0. (D) The power spectral density corresponding to (A) for different diffusion coefficient D=0. (E) Same as in (A) but for different diffusion coefficient D=0.00001. (F) Same as in (B) but for different diffusion coefficient D=0.00001. (G) Same as in (C) but for different diffusion coefficient D=0.00001. (H) Same as in (D) but for different diffusion coefficient D=0.00001. (I) Same as in (A) but for different diffusion coefficient D=0.00005. (J) Same as in (B) but for different diffusion coefficient D=0.00005. (K) Same as in (C) but for different diffusion coefficient D=0.00005. (L) Same as in (D) but for different diffusion coefficient D=0.00005. (M) Same as in (A) but for different diffusion coefficient D=0.0001. (N) Same as in (B) but for different diffusion coefficient D=0.0001. (O) Same as in (C) but for different diffusion coefficient D=0.0001. (P) Same as in (D) but for different diffusion coefficient D=0.0001.

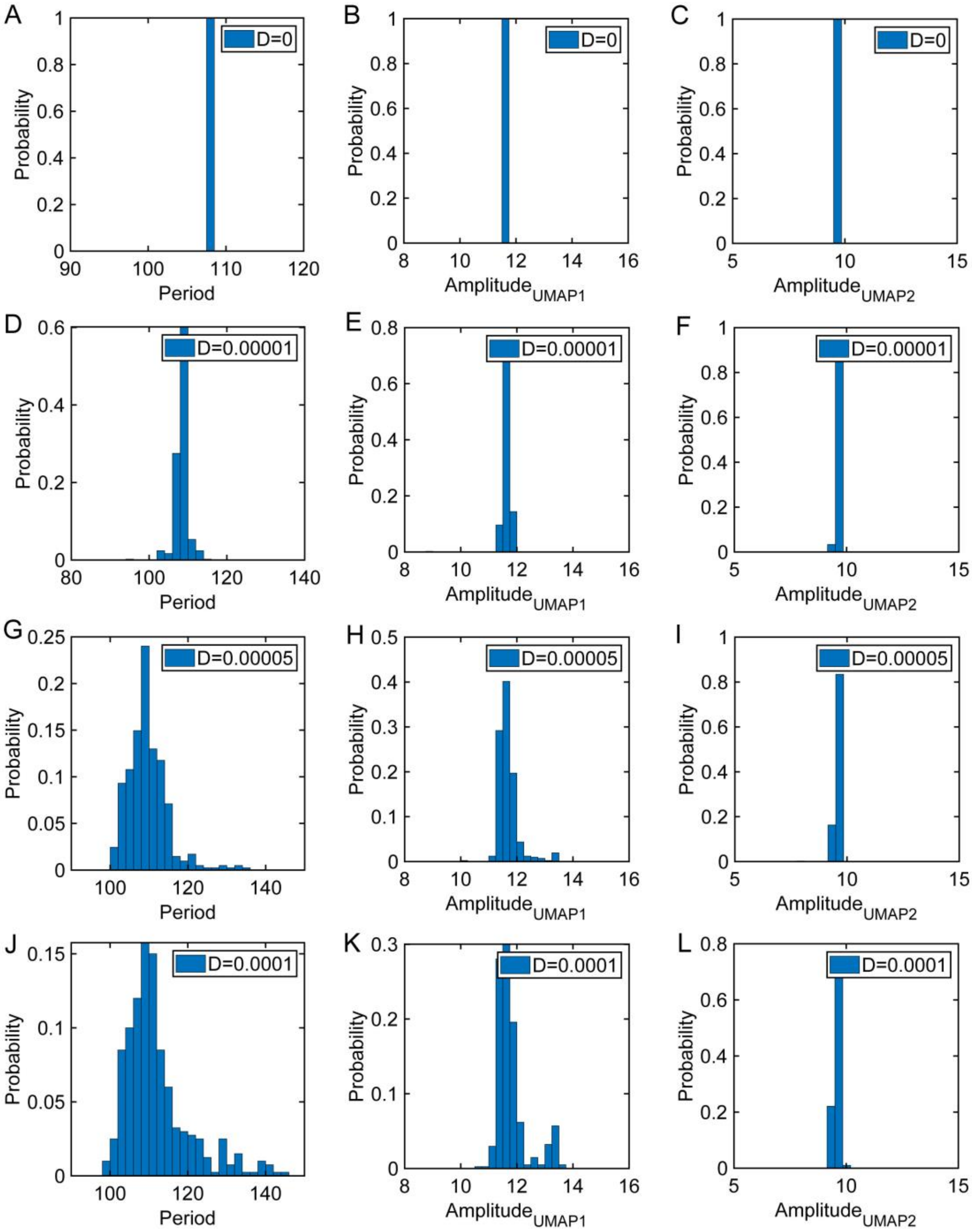

Figure S8. The period and amplitude distribution of cell cycle oscillation dynamics with different strength of noise. (A) The distribution of cell cycle period for different diffusion coefficient D=0. (B) The distribution of amplitude in UMAP1 for different diffusion coefficient D=0. (C) The distribution of amplitude in UMAP2 for different diffusion coefficient D=0. (D) Same as in (A) but for different diffusion coefficient D=0.00001. (E) Same as in (B) but for different diffusion coefficient D=0.00001. (F) Same as in (C) but for different diffusion coefficient D=0.00001. (G) Same as in (A) but for different diffusion coefficient D=0.00005. (H) Same as in (B) but for different diffusion coefficient D=0.00005. (I) Same as in (C) but for different diffusion coefficient D=0.00005. (J) Same as in (A) but for different diffusion coefficient D=0.0001. (K) Same as in (B) but for different diffusion coefficient D=0.0001. (L) Same as in (C) but for different diffusion coefficient D=0.0001.

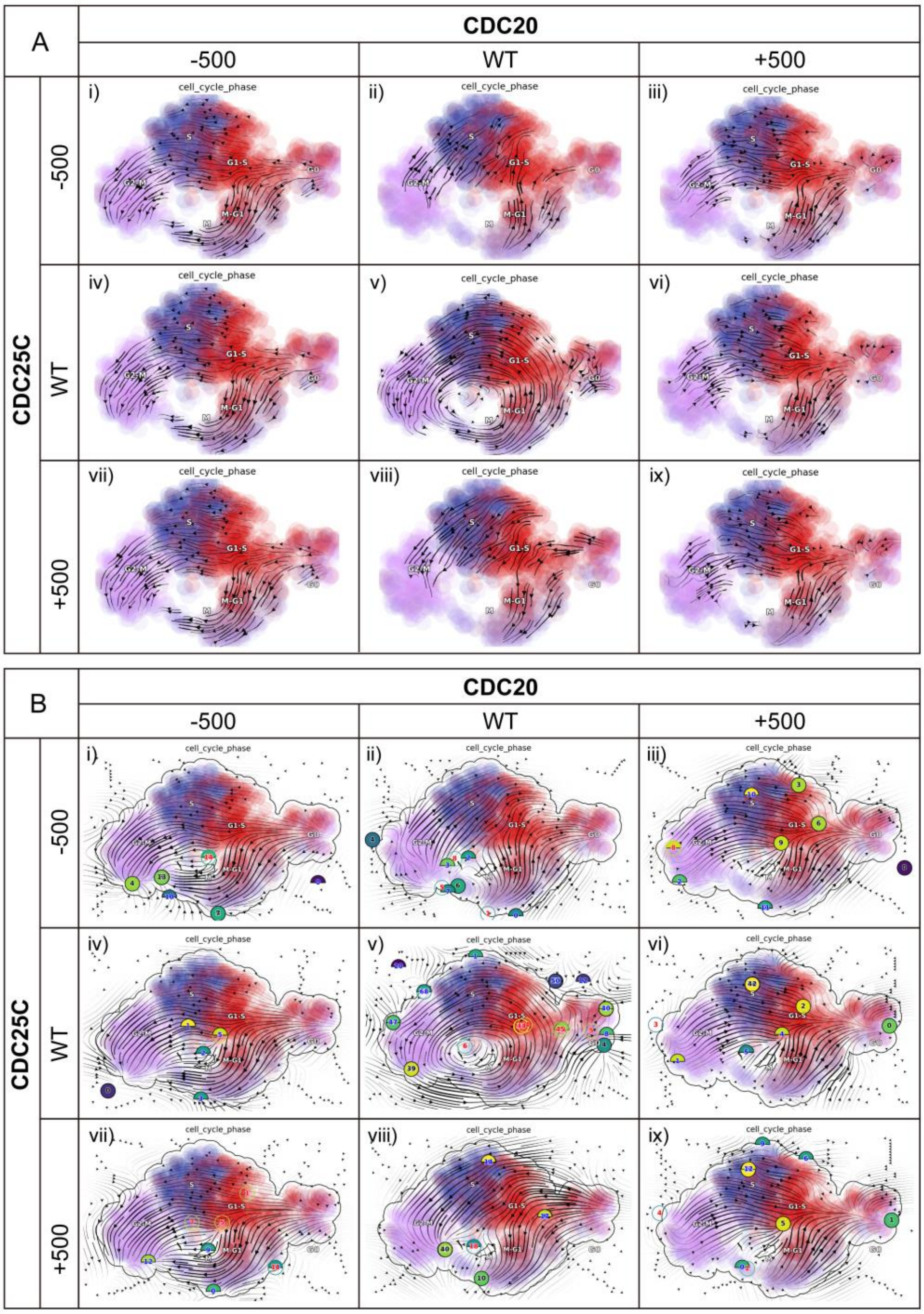

Figure S9. RNA velocity and reconstructed vector field of cell cycle under genetic perturbation. (A) *In silico* perturbation trajectory predictions: (i) Suppression of both *CDC20* and *CDC25C*. (ii) Suppression of *CDC25C* only. (iii) Activation of *CDC20* and suppression of *CDC25C*. (iv) Suppression of *CDC20* only. (v) WT. (vi) Activation of *CDC20* only. (vii) Suppression of *CDC20* and activation of *CDC25C*. (viii) Activation of *CDC25C* only. (ix) Activation of both *CDC20* and *CDC25C*.(B) *In silico* perturbation vector field predictions: (i) Suppression of both *CDC20* and *CDC25C*. (ii) Suppression of *CDC25C* only. (iii) Activation of *CDC20* and suppression of *CDC25C*. (iv) Suppression of *CDC20* only. (v) WT. (vi) Activation of *CDC20* only. (vii) Suppression of *CDC20* and activation of *CDC25C*. (viii) Activation of *CDC25C* only. (ix) Activation of both *CDC20* and *CDC25C*.

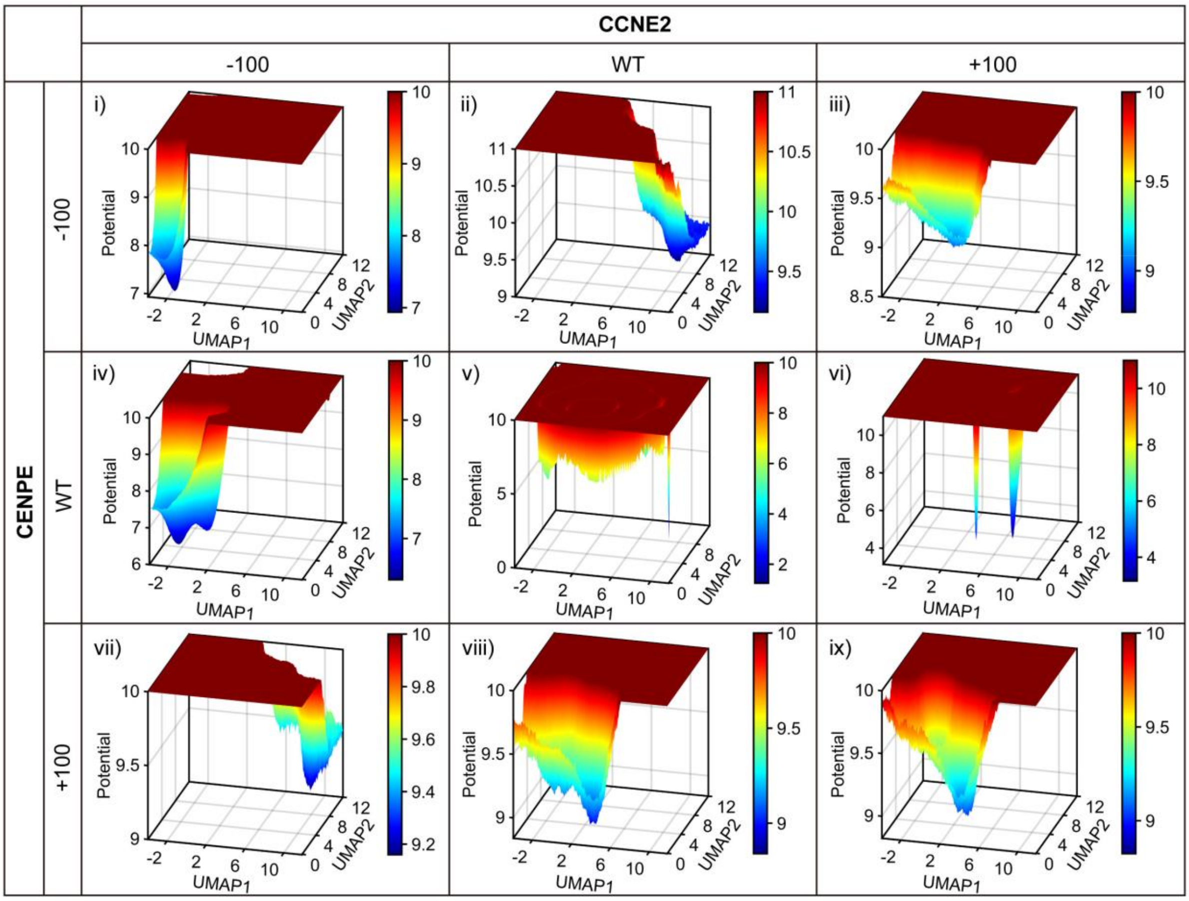

Figure S10. The genetic perturbation alters of U2OS cell cycle global dynamics. (A) Cell cycle global dynamics landscape of U2OS cells in UMAP with different genetic perturbation. (i) Suppression of both *CCNE2* and *CENPE*. (ii) Suppression of *CENPE* only. (iii) Activation of *CCNE2* and suppression of *CENPE*. (iv) Suppression of *CCNE2* only. (v) WT. (vi) Activation of *CCNE2* only. (vii) Suppression of *CCNE2* and activation of *CENPE*. (viii) Activation of *CENPE* only. (ix) Activation of both *CCNE2* and *CENPE*.

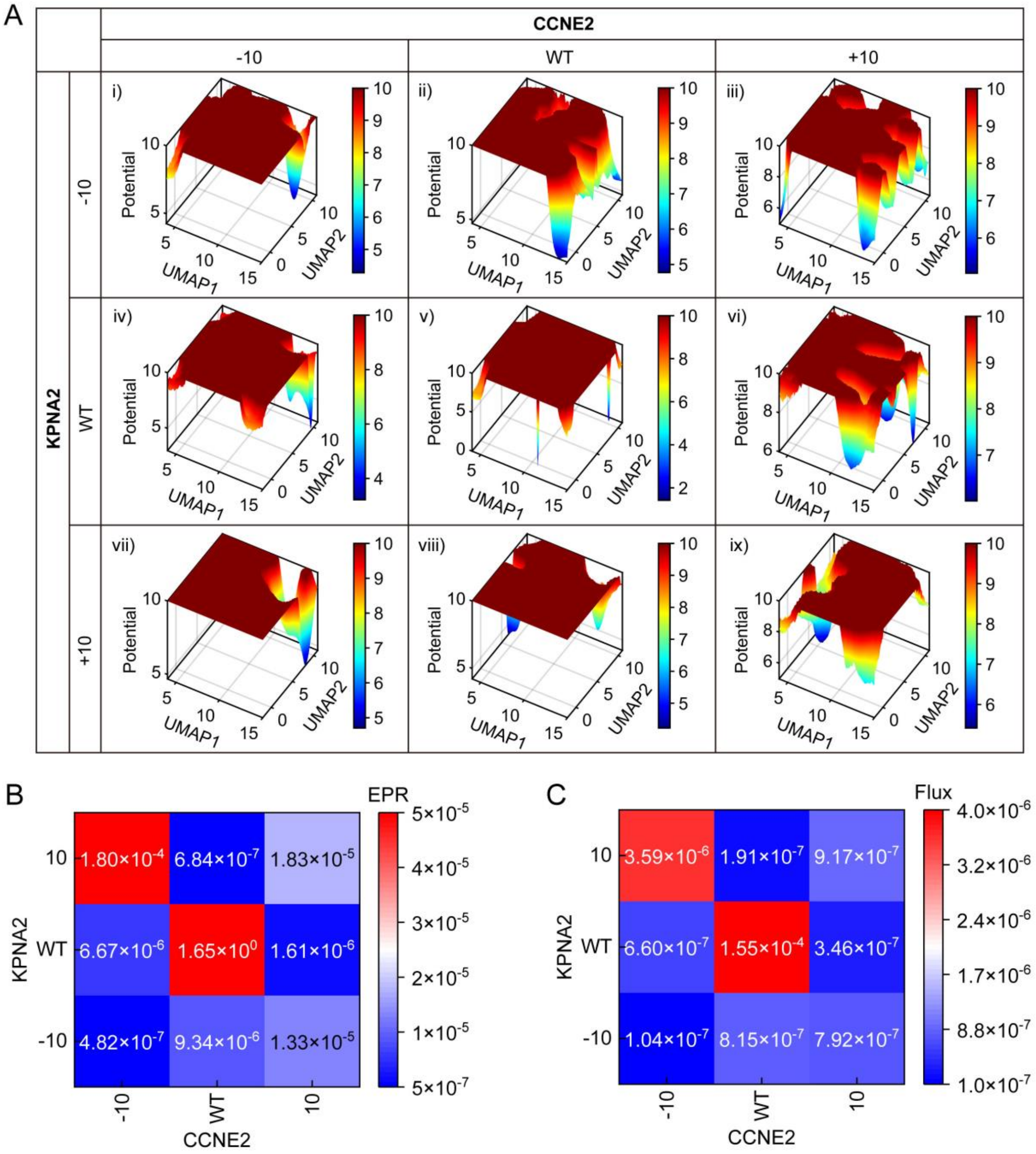

Figure S11. The genetic perturbation alters of RPE1 cell cycle landscape-flux. (A) Cell cycle global dynamics landscape of REP1 cells in UMAP with different genetic perturbation. (i) Suppression of both *CCNE2* and *KPNA2*. (ii) Suppression of *KPNA2* only. (iii) Activation of *CCNE2* and suppression of *KPNA2*. (iv) Suppression of *CCNE2* only. (v) WT. (vi) Activation of *CCNE2* only. (vii) Suppression of *CCNE2* and activation of *KPNA2*. (viii) Activation of *KPNA2* only. (ix) Activation of both *CCNE2* and *KPNA2*. (B) EPR of cell cycle nonequilibrium thermodynamics with different genetic perturbation. (C) The average Flux of cell cycle nonequilibrium dynamics with different genetic perturbation.

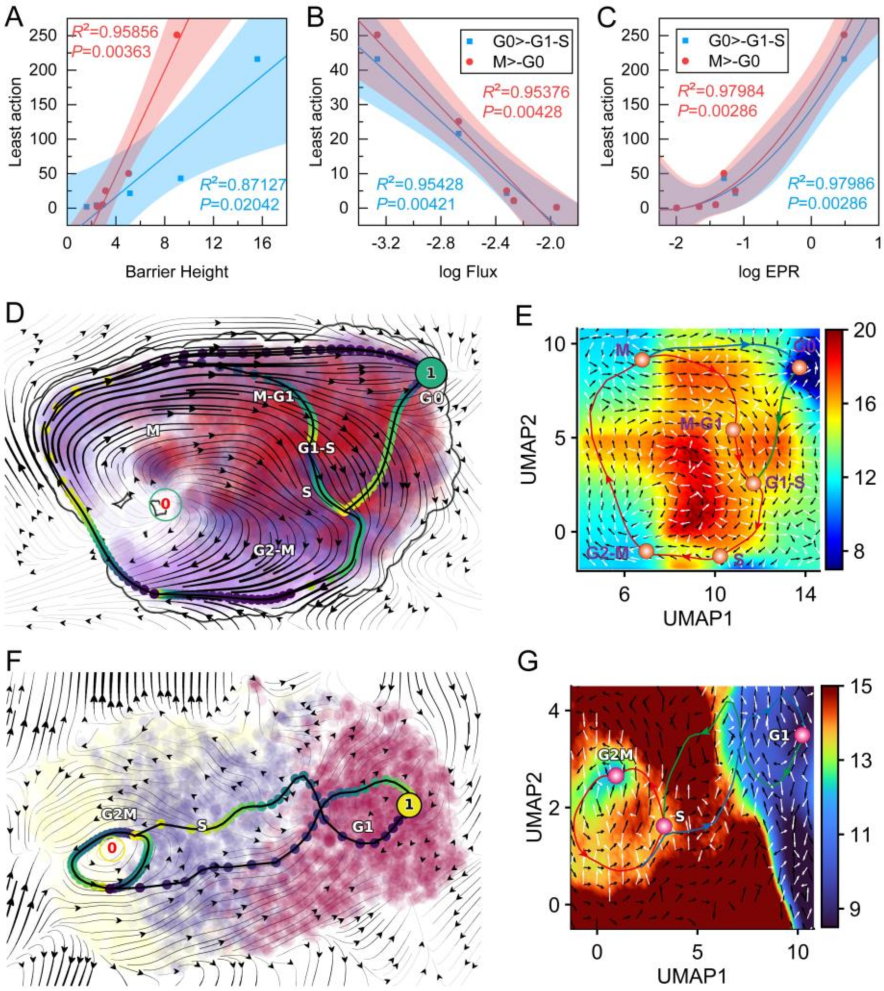

Figure S12. LAPs and MFPT in cell cycle initiation and termination. (A) The correlation between barrier height and least action when diffusion coefficients are changed. The line is a fitting correlation line, and the shaded part is the 95% fitting confidence interval. (B) The correlation between the logarithm of flux and least action when diffusion coefficients are changed. (C) The correlation between the logarithm of EPR and least action when diffusion coefficients are changed. (D) LAPs between different cell cycle phases in the velocity vector field of RPE1 cells. The color of digits in each node reflects the type of fixed point: red, emitting fixed point; black, absorbing fixed point. The color of the numbered nodes corresponds to the confidence of the fixed points. The color of the dots along the paths corresponds to the direction. (E) LAPs between different cell cycle phases in 2D landscape of RPE1 cells. The black arrows present the curl flux and the white arrows present the gradient force in the landscape. (F) LAPs between different cell cycle phases in the velocity vector field of human fibroblasts. The color of digits in each node reflects the type of fixed point: red, emitting fixed point; black, absorbing fixed point. The color of the numbered nodes corresponds to the confidence of the fixed points. The color of the dots along the paths corresponds to the direction. (G) LAPs between different cell cycle phases in 2D landscape of human fibroblasts. The black arrows present the curl flux and the white arrows present the gradient force in the landscape.

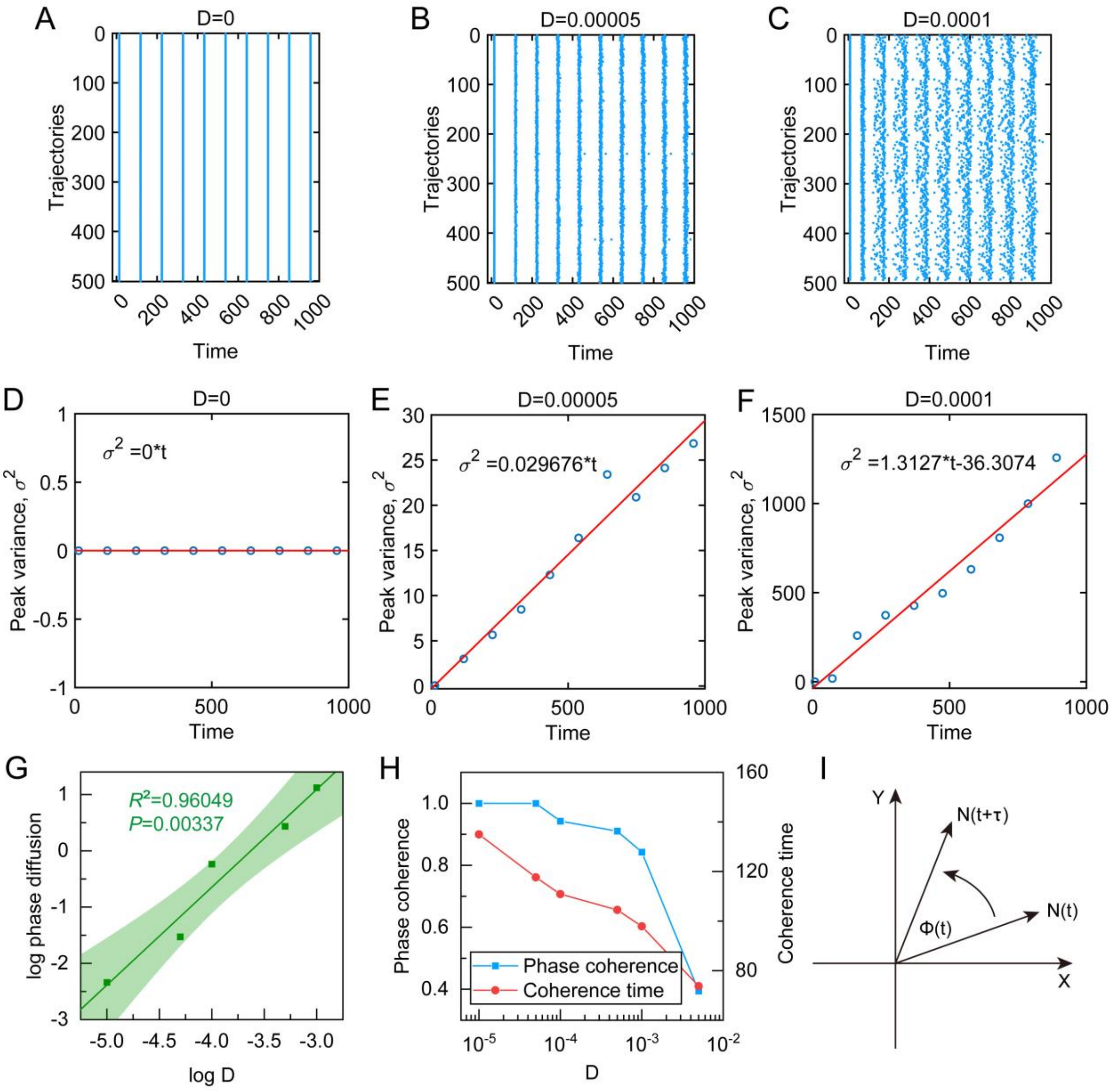

Figure S13. Phase diffusion and phase coherence of cell cycle oscillation with different noise. (A-C) Raster plot of the peak times for 500 different trajectories starting with the same initial condition at the different diffusion coefficient, D=0 (A), D=0.00005 (B) and D=0.0001 (C). (D-F) Peak time variance σ^2 goes linearly with the average peak time for the different diffusion coefficient, with the linear coefficient defined as the peak-time diffusion constant, D=0 (D), D=0.00005 (E) and D=0.0001 (F). (G) The logarithm of phase diffusion and the logarithm of diffusion coefficient D. The line is a fitting correlation line, and the shaded part is the 95% fitting confidence interval. (H) The change of coherence time and phase coherence when diffusion coefficients (fluctuations) are changed. (I) Sketch map for the definition of phase coherence.

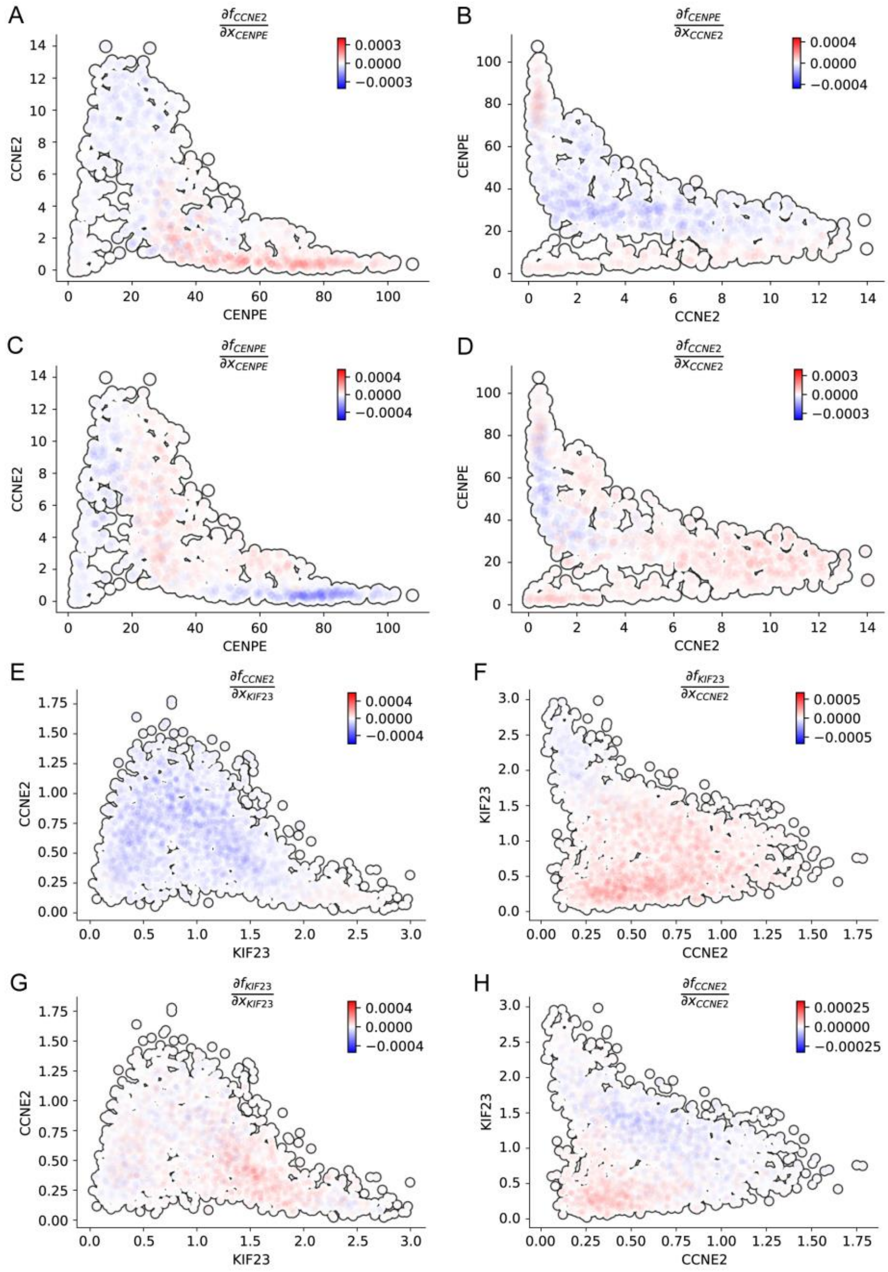

Figure S14. Jacobian analyses of the cell cycle regulatory interactions in the gene expression space. (A-D) Jacobian analyses of the U2OS cell cycle regulatory interactions in the CCNE2 and CENPE expression space. Activation from *CENPE* to *CCNE2* in the *CENPE* and *CCNE2* expression space (A), repression from *CCNE2* to *CENPE* in the *CCNE2* and *CENPE* expression space (B), (C) self-activation of *CENPE* in the *CENPE* and *CCNE2* expression space (C), and self-activation of *CCNE2* in the *CCNE2* and *CENPE* expression space (D). (E-H) Jacobian analyses of the U2OS cell cycle regulatory interactions in the CCNE2 and KIF23 expression space. Repression from *KIF23* to *CCNE2* in the *KIF23* and *CCNE2* expression space (E), activation from *CCNE2* to *KIF23* in the *CCNE2* and *KIF23* expression space (F), self-activation of *KIF23* in the *KIF23* and *CCNE2* expression space (G), and self-activation of *CCNE2* in the *CCNE2* and *KIF23* expression space (H).

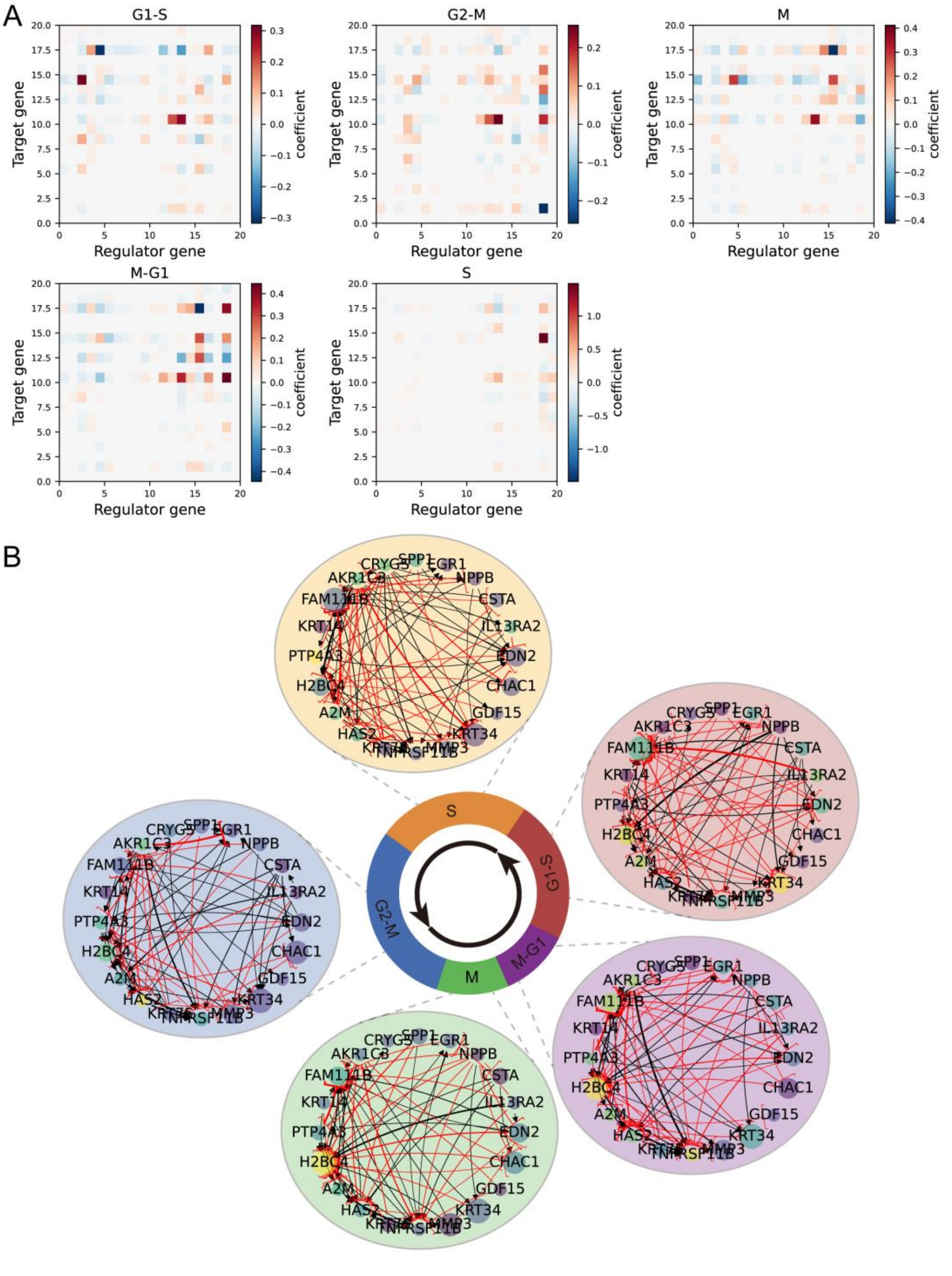

Figure S15. Inference of cell-cycle-phase specific gene regulatory networks for the U2OS cell cycle by single-cell transcriptomics data. (A) The cell-cycle-phase specific gene-gene interaction jacobian matrices of each cell cycle phase. The positive coefficients (red) imply an activation from the regulator gene (x-axis) to the target gene (y-axis), while negative coefficients (blue) imply inhibition. (B) The cell-cycle-phase specific gene regulatory networks. The node size is scaled based on gene expression in the specific cell type and the color of nodes represent the node centrality in the network, the black arrows represent the activation and the red arrows represent the inhibition, and the width of connection arrows is scaled based on the strength of gene interactions.

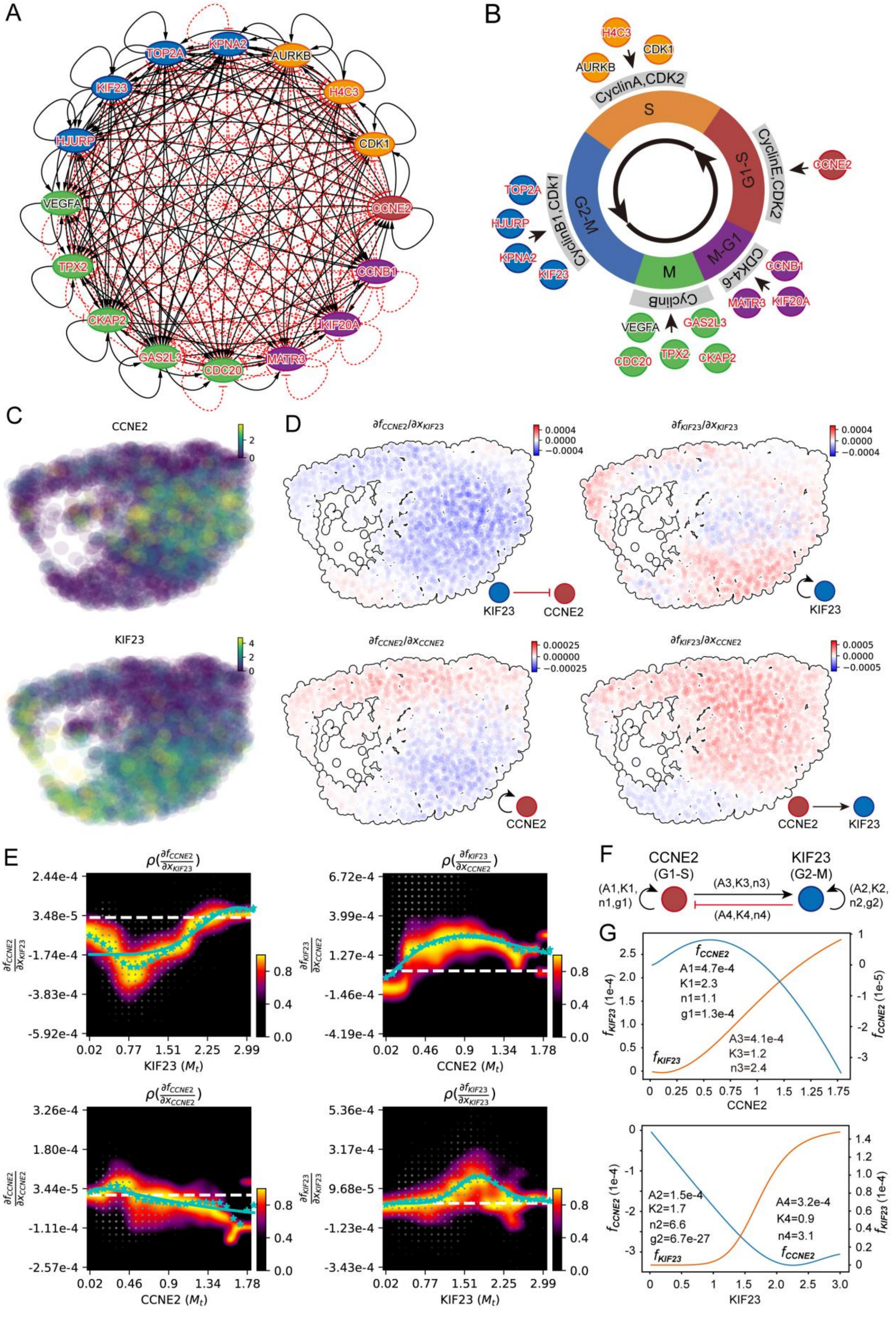
A
TOP2A
KPNA2
AURKB
KIF23
H4C3
HJURP
CDK1
VEGFA
CCNE2
TPX2
CCNB1
CKAP2
KIF20A
GAS2L3
CDC20
MATR3
B
CyclinA,CDK2
S
G1-S
CyclinE,CDK2
G2-M
CyclinB1,CDK1
M-G1
CDK4-6
M
CyclinB
C
CCNE2
KIF23
D
KIF23
CCNE2
E
KIF23 (M_t)
CCNE2 (M_t)
F
CCNE2
(G1-S)
KIF23
(G2-M)
(A1,K1,
n1,g1)
(A3,K3,n3)
(A4,K4,n4)
(A2,K2,
n2,g2)
G
A1=4.7e-4
K1=2.3
n1=1.1
g1=1.3e-4
A3=4.1e-4
K3=1.2
n3=2.4
A2=1.5e-4
K2=1.7
n2=6.6
g2=6.7e-27
A4=3.2e-4
K4=0.9
n4=3.1

Figure S16. Inference of gene regulatory networks for the RPE1 cell cycle by single-cell transcriptomics data. (A) The interaction network of genes of each cell cycle phase of RPE1 cells. All differentially expressed genes in each cell cycle phase are used to construct network; nodes (genes) with more than one edge are shown. Node colors represent different cycle phases (red: G1-S, yellow: S, blue: G2-M, green: M, purple: M-G1), the black arrows represent the activation and the red arrows represent the inhibition. Genes with black representing TF and red representing non-TFs. (B) The diagram for the cell cycle model with key genes of each cell cycle phase. (C) *CCNE2* has high expression in G1-S phase and *KIF23* has high expression in G2-M phase. (D) Molecular mechanisms underlying the maintenance of the cell cycle. (i) Repression of *CCNE2* by *KIF23*. (ii) Self-activation of *KIF23*. (iii) Self-activation of *CCNE2*. (iv) *CCNE2* activates *KIF23*. (E) Fitting the function of Jacobian versus gene expression with derivatives of a simplistic inhibitory or activation Hill equation. White dashed line corresponds to the zero Jacobian value. The blue stars at each x axis grid point correspond to the weighted mean of the Jacobian values for that point. The blue solid lines are the resultant fittings for the Jacobian. (F) Schematic summarizing the interactions involving *CCNE2* and *KIF23*. (G) The velocity kinetic curves over gene expression changes of the corresponding fitted Hill equations of (E).

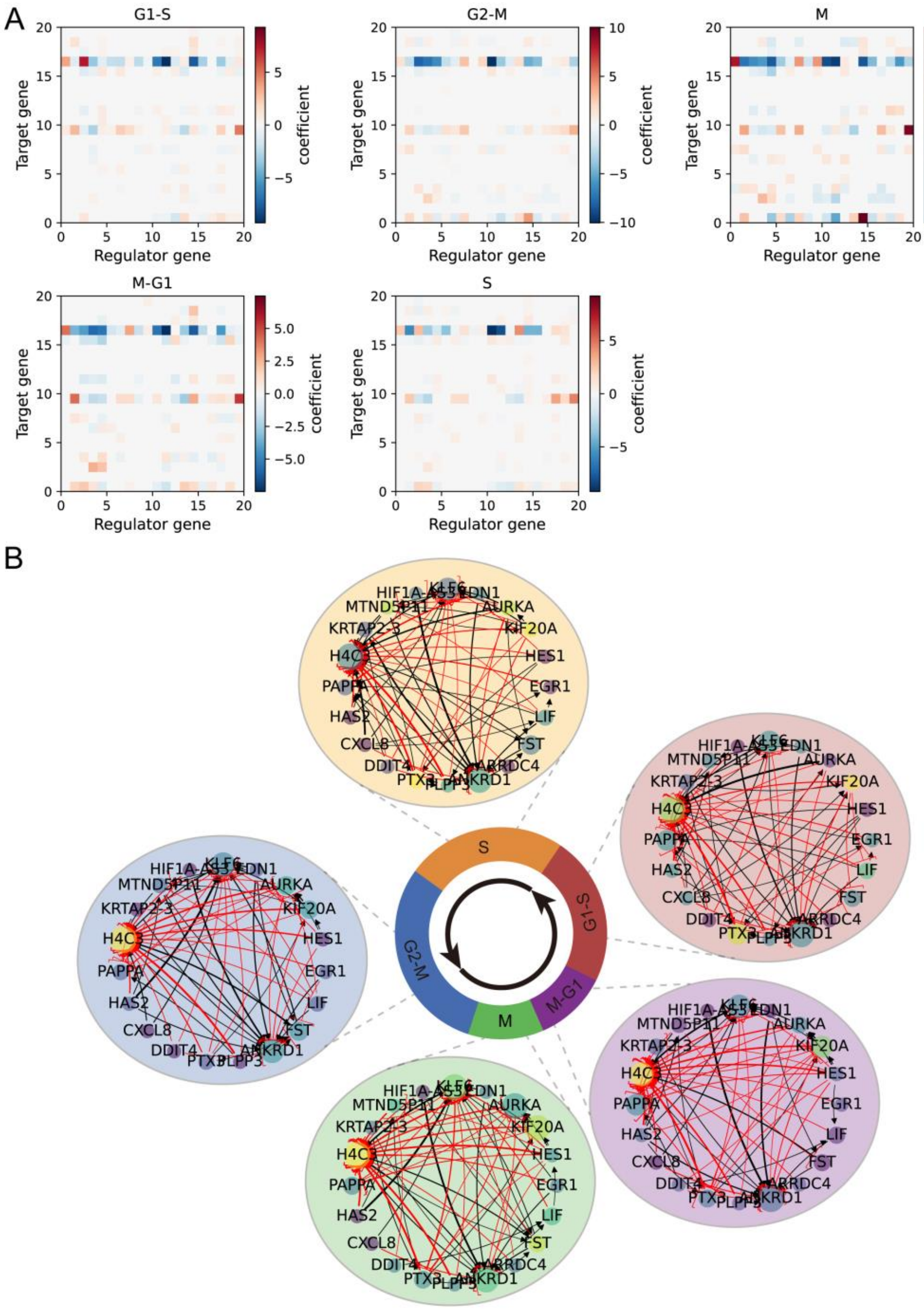

Figure S17. Inference of cell-cycle-phase specific gene regulatory networks for the RPE1 cell cycle by single-cell transcriptomics data. (A) The cell-cycle-phase specific gene-gene interaction jacobian matrices of each cell cycle phase. The positive coefficients (red) imply an activation from the regulator gene (x-axis) to the target gene (y-axis), while negative coefficients (blue) imply inhibition. (B) The cell-cycle-phase specific gene regulatory networks. The node size is scaled based on gene expression in the specific cell type and the color of nodes represent the node centrality in the network, the black arrows represent the activation and the red arrows represent the inhibition, and the width of connection arrows is scaled based on the strength of gene interactions.
